# Nucleotide‐binding leucine‐rich repeat network underlies nonhost resistance of pepper against the Irish potato famine pathogen *Phytophthora infestans*

**DOI:** 10.1111/pbi.14039

**Published:** 2023-03-13

**Authors:** Soohyun Oh, Sejun Kim, Hyo‐Jeong Park, Myung‐Shin Kim, Min‐Ki Seo, Chih‐Hang Wu, Hyun‐Ah Lee, Hyun‐Soon Kim, Sophien Kamoun, Doil Choi

**Affiliations:** ^1^ Plant Immunity Research Center, College of Agriculture and Life Sciences Seoul National University Seoul South Korea; ^2^ Department of Agriculture, Forestry and Bioresources, Plant Genomics and Breeding Institute, College of Agriculture and Life Sciences Seoul National University Seoul South Korea; ^3^ The Sainsbury Laboratory University of East Anglia, Norwich Research Park Norwich UK; ^4^ Department of Horticulture, Division of Smart Horticulture Yonam University Cheonan South Korea; ^5^ Korean Research Institute of Bioscience & Biotechnology (KRIBB) Daejeon South Korea; ^6^ Present address: Institute of Plant and Microbial Biology, Academia Sinica Taipei Taiwan

**Keywords:** nonhost resistance, Solanaceae, potato late blight, nucleotide‐binding leucine‐rich repeats network, robustness

## Abstract

Nonhost resistance (NHR) is a robust plant immune response against non‐adapted pathogens. A number of nucleotide‐binding leucine‐rich repeat (NLR) proteins that recognize non‐adapted pathogens have been identified, although the underlying molecular mechanisms driving robustness of NHR are still unknown. Here, we screened 57 effectors of the potato late blight pathogen *Phytophthora infestans* in nonhost pepper (*Capsicum annuum*) to identify avirulence effector candidates. Selected effectors were tested against 436 genome‐wide cloned pepper NLRs, and we identified multiple functional NLRs that recognize *P. infestans* effectors and confer disease resistance in the *Nicotiana benthamiana* as a surrogate system. The identified NLRs were homologous to known NLRs derived from wild potatoes that recognize *P. infestans* effectors such as Avr2, Avrblb1, Avrblb2, and Avrvnt1. The identified Ca*Rpi‐blb2* is a homologue of *Rpi‐blb2*, recognizes Avrblb2 family effectors, exhibits feature of lineage‐specifically evolved gene in microsynteny and phylogenetic analyses, and requires pepper‐specific NRC (NLR required for cell death)‐type helper NLR for proper function. Moreover, *CaRpi‐blb2*–mediated hypersensitive response and blight resistance were more tolerant to suppression by the PITG_15 278 than those mediated by *Rpi‐blb2*. Combined results indicate that pepper has stacked multiple NLRs recognizing effectors of non‐adapted *P. infestans*, and these NLRs could be more tolerant to pathogen‐mediated immune suppression than NLRs derived from the host plants. Our study suggests that NLRs derived from nonhost plants have potential as untapped resources to develop crops with durable resistance against fast‐evolving pathogens by stacking the network of nonhost NLRs into susceptible host plants.

## Introduction

Plants are resistant to most of the surrounding pathogenic microorganisms. This plant immune response against non‐adapted pathogens is known as nonhost resistance (NHR) (Heath, 2000). NHR is durable and by definition is hardly overcome by the pathogen. Subsequently, understanding the molecular mechanisms of NHR could provide promising strategies for crop protection. Various plant defence mechanisms including pre‐ and post‐invasion defence have been reported to be associated with NHR (Lee *et al*., 2017; Lipka *et al*., 2005). Among the multiple layers of plant immune strategies associated with NHR, cytoplasmic immune receptor proteins, known as nucleotide‐binding leucine‐rich repeats (NLRs), have been suggested as components of NHR particularly in recently diverged plant species (Schulze‐Lefert and Panstruga, 2011).

NLRs constitute one of the largest gene families in plants and generally recognize pathogen effectors that are secreted into plant cells to modulate plant defence mechanisms and enhance pathogen proliferation (Dodds and Rathjen, 2010). NLRs directly or indirectly recognize effectors and subsequently initiate defence signalling to suppress pathogen growth; these events often result in the hypersensitive cell death response (HR) (Cui *et al*., 2015). NLRs have been implicated in NHR because various effectors induce HR in nonhost plants. In a few cases, the corresponding NLRs have been identified and successfully transferred into the host plants to confer resistance (Lee *et al*., 2017; Oh and Choi, 2022). For example, the Arabidopsis NLR *WRR4* confers resistance against the non‐adapted pathogen *Albugo candida* when transferred into the susceptible hosts *Brassica juncea* and *B. napus* (Borhan *et al*., 2008, 2010). Transfer of the maize NLR *Rxo1* into rice conferred resistance against *Xanthomonas oryzae* pv. *oryzicola*, which causes bacterial streak on rice (Zhao *et al*., 2004, 2005). These results suggest that NLRs could be components of NHR. This concept is intriguing because NLR‐mediated resistance is notorious for being relatively unstable due to the emergence of virulent pathogen races. Thus, the contributions of NLR to NHR remain to be determined.

The oomycete *P. infestans* causes the most destructive potato disease, as evidenced by the Irish potato famine (Kamoun *et al*., 2015). This oomycete pathogen is armed with hundreds of RXLR effectors (defined by a conserved N‐terminal Arg‐Xaa‐Leu‐Arg motif) that can be secreted into host cells (Cooke *et al*., 2012; Haas *et al*., 2009) and has a relatively narrow host range among the Solanaceae (Fry, 2008; Lee *et al*., 2014). Although more than 20 functional NLRs have been identified in wild *Solanum* species and transferred into potato, most of them have been repeatedly overcome by virulent isolates of *P. infestans* (Anderson *et al*., 2015; Haverkort *et al*., 2016).

Our previous study reported that multiple RXLR effectors of *P. infestans* induce HR‐like cell death in the nonhost pepper plant (*C. annuum*), and several of these effector‐mediated cell death phenotypes were segregated according to Mendelian inheritance (Lee *et al*., 2014). We concluded that multiple genetic factors recognizing *P. infestans* effectors may underpin NHR of pepper against *P. infestans*. Most NLRs derived from wild *Solanum* species that are closely related to the original host of *P. infestans* tend to be rapidly overcome by virulent races of the pathogen (Förch *et al*., 2010; Vleeshouwers *et al*., 2011). Therefore, identification of pepper NLRs that respond to *P. infestans* could advance our understanding on the robustness of NHR and provide strategies for developing durable resistance of crops.

## Results

### Multiple *P. infestans* effectors trigger cell death on nonhost pepper

**Figure 1:**
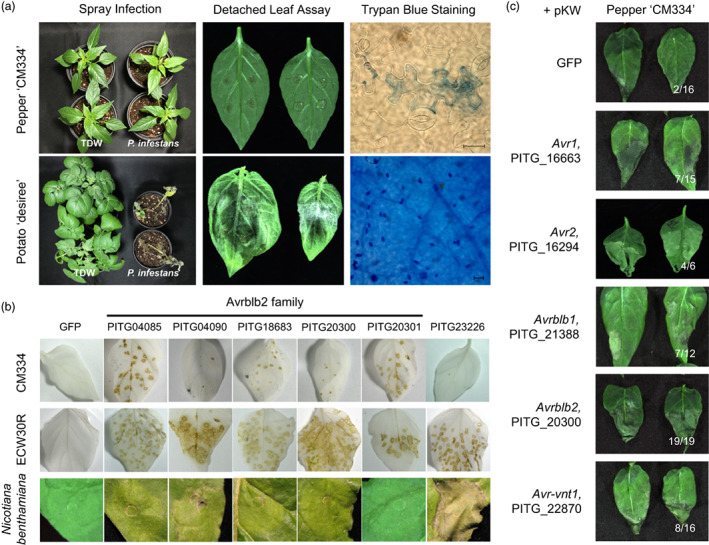
*Phytophthora infestans* effectors triggered HR‐like cell death on nonhost pepper. (a) Pepper (cv. CM334) and potato (cv. Desiree) were inoculated with *P. infestans* T30‐4 (*Pi* T30‐4) zoospores or distilled water (TDW) as a negative control. HR cell death and sporangia development were visualized with trypan blue staining. Photographs and microscopic images were taken at 6 days post inoculation (dpi). Bars = 50 μm. (b) Among the 57 *Pi* effectors, PVX virion–mediated expression of Avrblb2 family effectors (PITG_20300, 04085, 04090, 18683, and 20301) along with PITG_23226 triggered cell death on CM334 and ECW30R pepper cultivars. Only PITG_23226 also triggered cell death in *N. benthamiana* via Agroinfiltration. GFP was included as a negative control in both experiments. The observed phenotypes were consistent in the three biological replicates. (c) Twenty‐five *Pi* effectors were expressed via Agroinfiltration on CM334 leaves. The numbers of cell death/replicated cases are presented on the lower right of each image. Multiple effectors known for its corresponding *Solanum* NLRs triggered cell death on CM334 leaves compared to *GFP*‐expressed leaves.

Pepper responds against the invasion of *P. infestans* with a highly localized cell death which is phenotypic hallmark of NLR‐mediated resistance (Figure 1a). To identify pepper NLRs that recognize *P. infestans* effectors, we tested whether effector candidates triggered HR on nonhost pepper plants using the *potato virus X* (PVX)–mediated gene expression system (Lee *et al*., 2014; Vleeshouwers *et al*., 2011). We selected 57 core RXLR effectors that are conserved across four *P. infestans* isolates (T30‐4, NL07434, P17777, and 06_3928A) and expressed within 2–3 days after infecting potato (Cooke *et al*., 2012). Recombinant PVX virions expressing each RXLR effector were rubbed onto the leaves of two pepper accessions, Criollo de Morelos 334 (CM334) and Early California Wonder (ECW). Six of the 57 effectors consistently induced cell death on both pepper accessions (Figure 1b; Table S1). By contrast, PVX‐GFP did not cause any specific symptoms although GFP fluorescence was detected in CM334 and ECW (Figure S1). Five of the six effectors were Avrblb2 (PITG_20300) and its paralogs (PITG_04085, 04090, 18683, 20301) (Oh *et al*., 2009), and none of these effectors caused cell death in *N. benthamiana* (Figure 1b). Although several effectors were identified from the PVX screening, the low GFP fluorescence in CM334 indicated that PVX had lower proliferation in CM334 than in ECW (Figure S1), and the effectors triggered much lower levels of cell death in CM334 than in ECW (Figure 1b; Table S1). Therefore, we also screened effectors using Agrobacterium‐mediated transient expression in CM334 and identified additional cell death–inducing *P. infestans* effectors, including Avr2 (Saunders *et al*., 2012), Avrblb1 (Vleeshouwers *et al*., 2008), and Avrvnt1 (Pel, 2010), which triggered a more intensive cell death phenotype than that observed in GFP‐expressing leaves (Figures 1c, S2). These results indicate that NLR‐mediated recognition of multiple effectors could underlie NHR of pepper against *P. infestans*, consistent with a previous study (Lee *et al*., 2014). The candidate effectors Avr1, Avr2, Avrblb1, Avrblb2, and Avrvnt1 were already known for the corresponding NLRs derived from wild *Solanum* species. Although the corresponding *Solanum* NLRs (R1, R2, Rpi‐blb1, Rpi‐blb2, and Rpi‐vnt1) were reportedly overcome by multiple *P. infestans* isolates (Förch *et al*., 2010), none of those isolate can infect pepper in natural condition. Therefore, we tried to identify pepper NLRs that recognize candidate *Pi* effectors and investigate how pepper NLRs contributed to the robust NHR against *P. infestans*.

### Multiple pepper NLRs recognize *P. infestans* effectors and confer resistance in *N. benthamiana*

**Figure 2:**
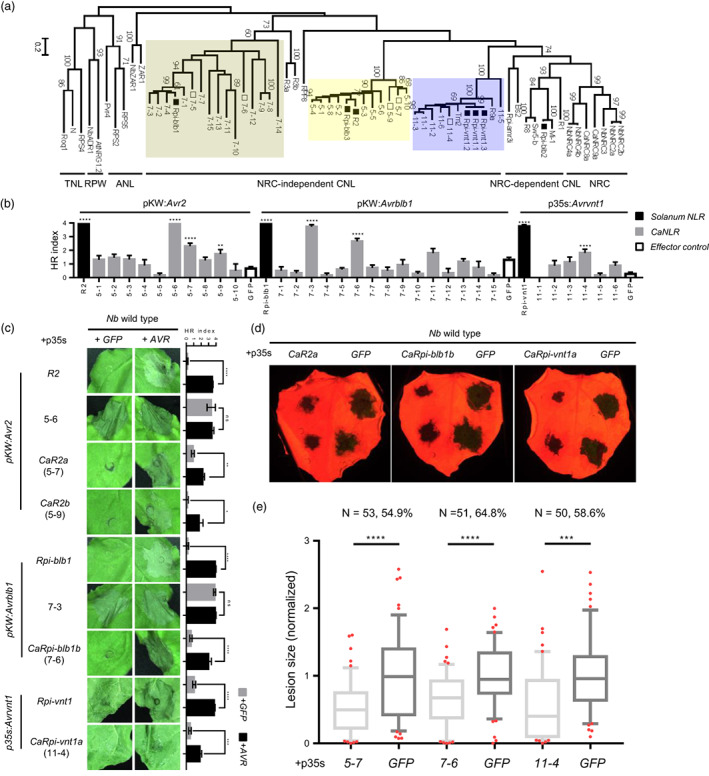
Multiple pepper NLRs homologous to R2, Rpi‐blb1, and Rpi‐vnt1 trigger HR against Avr2, Avrblb1, and Avrvnt1 and confer resistance to *P. infestans* in *N. benthamiana*. (a) Phylogenetic analysis of known R genes with CaNLR homologues. The tested CaNLR clades are coloured with boxes. Reference R genes and identified CaNLRs are marked with black and white rectangles at the end of nodes. (b) HR intensities of the 10, 15, and 6 pepper NLRs that are homologous to corresponding R genes of Avr2 (R2), Avrblb1 (Rpi‐blb1), and Avrvnt1 (Rpi‐vnt1), respectively, when co‐expressed with each effector. Several NLRs exhibited significant cell death phenotypes, marked with an asterisk (*), against each corresponding effector compared to GFP‐expressed cases as a negative control (white bars). Statistical significance was analysed using the unpaired *t*‐test (error bars indicate SEM from at least three replicates for non‐significant cases and at least 10 replicates for significant cases; **P < 0.005; ****P < 0.0001). (c) Representative images and HR intensity graphs of identified CaNLRs. CaNLR5‐6 and CaNLR7‐3 exhibited autoactive cell death when expressed with *GFP* in *N. benthamiana*. Statistical significance was analysed using the unpaired *t*‐test (error bars indicate SEM from at least four replicates; ns, nonsignificant; *P < 0.05; **P < 0.005; ***P < 0.0005; ****P < 0.0001). (d) Expression of *CaNLR5‐7* (CA04g17640), *CaNLR7‐6* (CA01g31430), and *CaNLR11‐4* (CA03g00800) consistently reduced lesion size of *P. infestans* in *N. benthamiana* compared to *GFP*‐expression in the other half of the same leaves. (e) Average lesion sizes measured in three independent experiments are presented with 10–90 percentile box plots. Relative lesion sizes compared to those in *GFP*‐expression in the other half of the same leaves are marked with percentages on the top of each graph. Statistical significance was analysed using the unpaired *t*‐test (***P < 0.0005; ****P < 0.0001).

To identify pepper NLRs that respond to candidate effectors, we used a reverse genetics approach with *N. benthamiana* as a surrogate system (Shibata *et al*., 2010) rather than generating multiple populations by crossing pepper accessions with different cell death phenotype profiles against *Pi* effectors. For this screening, 426 NLRs (containing at least four major and minor motifs of the NB‐ARC domain) were cloned from pepper genomic DNA based on the pepper genome version 1.55 (Kim *et al*., 2014; Lee *et al*., 2021; Seo *et al*., 2016) (Tables S2, S3). These pepper NLRs were co‐expressed with each candidate effector on *N. benthamiana* leaves (1:1 via Agroinfiltration). Especially, Avr1, Avr2, Avrblb1, Avrblb2, and Avrvnt1 effectors are already known for the corresponding *Solanum* NLRs. Therefore, pepper NLRs homologous to R1 (CNL‐G3) (Ballvora *et al*., 2002), R2 (CNL‐G5) (Lokossou *et al*., 2009), Rpi‐blb1 (CNL‐G7) (Van Der Vossen *et al*., 2003; Vleeshouwers *et al*., 2008), Rpi‐blb2 (CNL‐G1) (Van Der Vossen *et al*., 2005), and Rpi‐vnt1 (CNL‐G11) (Foster *et al*., 2009; Pel *et al*., 2009) were tested with each corresponding effector (Figures 2a, S3; Table S3).

Multiple pepper NLRs induced cell death in response to Avr2, Avrblb1, and Avrvnt1 when co‐expressed in *N. benthamiana* (Figures 2b,c, S3, S4; Tables S3, S4). However, CaNLR5‐6 and 7–3 also exhibited cell death phenotypes without effectors (Figure 2c). Then we selected total 12 CaNLRs: four exhibited clear cell death (5‐9, and, 11‐4); four exhibited weak cell death (7‐11, and, 11‐6); and four did not exhibit cell death (7‐9, and, 11‐2) against corresponding effectors. We tested whether these 12 CaNLRs conferred resistance to *P. infestans* in *N. benthamiana* (Figures 2b, S5). As a result, a total of six CaNLRs conferred resistance to *P. infestans* in *N. benthamiana*, including all candidates exhibited clear cell death from the 1:1 screening (Figure S5). Especially, CaNLR5‐7, 7–6, and 11‐4 (designated as CaR2a, CaRpi‐blb1b, and CaRpi‐vnt1a, respectively) consistently reduced the lesion size in *N. benthamiana* compared to *GFP*‐expression in the other half of the same leaf (Figures 2d,e, S5). Similar with the lesion size data, expression level of *P. infestans* actin was also significantly decreased CaNLR‐expressed region of leaves supporting the identified CaNLRs contribute to resistance against *P. infestans* (Figures S6, S7). Unexpectedly, CaNLR7‐5 (CaRpi‐blb1a), which did not induce cell death in response to Avrblb1, also conferred resistance to *P. infestans*. We assumed that CaRpi‐blb1a may recognize another effector homologous to Avrblb1, or sharing similar virulence mechanisms. These combined results indicate that pepper possesses multiple NLRs recognizing *P. infestans* effectors, and these NLRs would contribute to NHR of pepper against *P. infestans*.

### Failure to identify pepper NLRs recognizing Avrblb2s via heterologous expression of single NLRs

Multiple pepper NLRs recognizing *P. infestans* effectors (Avr2, Avrblb1, and Avrvnt1) were identified from the 1:1 co‐expression screening. However, we did not observe cell death phenotypes using other effectors, even after co‐infiltrating with whole sets of 426 cloned pepper NLRs. One of these effectors was Avrblb2, which consistently induced HR‐like cell death in pepper (Figure 1b) but not in *N. benthamiana*. The Avrblb2 family could be one of the most important effectors because it is well conserved among most of *P. infestans* isolates and even several phylogenetically‐related *Phytophthora* species (Oliva *et al*., 2015). Therefore, we improved the 1:1 screening to identify pepper NLRs that recognize Avrblb2s.

The failure to identify pepper NLRs that recognize Avrblb2s could be due to a genetic background disparity between pepper and *N. benthamiana*. A class of Solanaceae NLRs called NRC (NLRs required for cell death) was proposed to form receptor networks with sensor NLRs (Wu *et al*., 2017). Sensor NLRs such as Rpi‐blb2, R1, R8, Rx, and Bs2 are dependent on NRC‐type helper NLRs for proper function, and Rpi‐blb2 requires NbNRC4 (Wu *et al*., 2017). We postulate that the sensor NLRs of pepper that recognize Avrblb2s are NRC‐dependent and functionally incompatible with NbNRCs. In this case, co‐transfer of the candidate sensor NLR with a pepper NRC (CaNRC) would be required to confer effective response to Avrblb2s in *N. benthamiana*.

### Pepper evolved lineage‐specific NRC4 homologues

**Figure 3:**
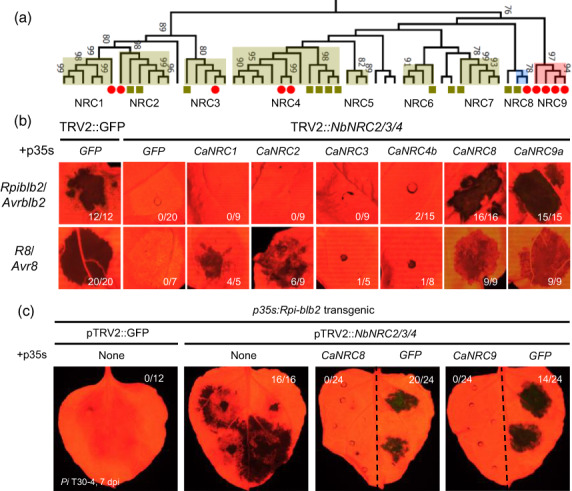
Pepper have evolved lineage‐specific NRCs that complement *NbNRC4* function. (a) Phylogenetic analysis of CNL‐G8 NLRs (NRC clade) of four Solanaceae species (tomato, potato, pepper, and *N. benthamiana*). Pepper and *N. benthamiana* genes are marked with a red circle and green rectangle, respectively. (b) Expression of *CaNRC* candidates restored HR phenotypes of NRC‐dependent sensor NLRs (Rpi‐blb2 and R8) against corresponding AVR effectors in *NbNRC2/3/4*‐silenced *N. benthamiana*. The number on the lower right of each image indicates the ratio of HR‐restored cases/total replications of each combination. Cell death phenotypes were visualized at 5 dpi using a Fluorescence *in vivo* imaging system (FOBI, CELLGENTEK). (c) Expression of *CaNRC8* or *CaNRC9a* at 24 h before *Pi* T30‐4 inoculation restored *Rpi‐blb2*‐mediated resistance in *NbNRC2/3/4*‐silenced *N. benthamiana*. The number on the upper right of each image indicates the cases of growing lesions/total inoculation sites, respectively. Photographs were taken at 6 dpi.

To identify *CaNRC*s, we performed phylogenetic analyses using NLRs of four Solanaceae plants (potato, tomato, pepper, and *N. benthamiana*). As described in Wu *et al*. (2017), we observed a distinct clade that included all functionally characterized NRCs such as NbNRC2/3/4 and SlNRC1 (Gabriëls *et al*., 2006; Wu *et al*., 2017), clustered with NRC‐dependent sensors (NRC‐S) that likely expanded from an ancestral NLR pair (Adachi *et al*., 2019; Figures 3a, S8). This NRC clade of CaNRC candidates was designated as CNL‐G8 (Seo *et al*., 2016). All *CaNRC* candidates were expressed at significant levels in pepper leaf transcriptome data, except for an NRC4 homologue of pepper (Ca06g12190) (Kim *et al*., 2018; Figure S9b). The NRC clade exhibited a clear bipartite structure divided into two subclades comprised of NRC1/2/3 and NRC4 homologues. NRC1/2/3 homologues were generally conserved throughout the four Solanaceae species. By contrast, NRC4 homologues exhibited species diversity (Figures 3a, S8), suggesting that each Solanaceae species independently evolved NRC4‐like genes after speciation. We named the unclassified NRC4‐like subclades NRC6 to NRC9 (Gabriëls *et al*., 2006; Wu *et al*., 2017, 2020). The NRC9 clade only contained pepper NLRs (Figures 3a, S8).

Subsequently, we performed genetic complementation assays to test which CaNRC candidates could restore HR phenotypes by complementing NbNRCs. Each of the CaNRC candidates was transiently co‐expressed with the known NRC‐S NLRs, *Rpi‐blb2* and *R8* (Jo, 2013; Vossen *et al*., 2016), which are *NRC4*‐ and *NRC2/3/4*‐dependent (Wu *et al*., 2017), respectively, and their corresponding AVR effectors in *NbNRC2/3/4*‐silenced *N. benthamiana* (Figure S10). Of the 10 CaNRC candidates, expression of *CaNRC1*, *2*, *8*, and *9a* restored the HR phenotypes of *R8* and expression of *CaNRC8* and *9a* restored Rpi‐blb2‐mediated HR (Figure 3b). Considering R8 is *NRC2/3/4*‐dependent and Rpi‐blb2 is *NRC4*‐dependent (Wu *et al*., 2017), the complementation results indicated that CaNRC1/2 and CaNRC8/9a had similar sensor NLR specificities with phylogenetically related NbNRCs, such as NRC2/3 and NRC4, respectively.

Although *NbNRC4*, *SlNRC4*, and *StNRC4* are highly expressed, contain intact domains of NLR (containing CC, NB‐ARC, and LRR domains), and were previously reported as functional (Wu *et al*., 2017, 2020), we could not detect intact *CaNRC4a/b* transcripts (CaNRC4a was not expressed and CaNRC4b was expressed without the CC domain) in qRT‐PCR assays (Figure S11). In addition, while expression of *CaNRC8* (Ca03g03390) or *CaNRC9a* (Ca11g01460) restored the *Rpi‐blb2*‐mediated HR phenotypes to *Avrblb2* and resistance to *P. infestans* by complementing *NbNRC4s*, expression of *CaNRC4b* (Ca11g02410) did not restored HR cell death (Figure 3b,c). These results indicate that pepper evolved functionally similar *NRC4*‐like helper NLRs, but these lineage‐specific CaNRCs have followed distinct evolutionary trajectories compared to their *N. benthamiana* homologues. This could lead to functional incompatibility between pepper NRC‐S NLRs and *NbNRCs*.

### Reverse genetics approach enables identification of a pepper NLR, CaRpi‐blb2a, that recognizes Avrblb2s

**Figure 4:**
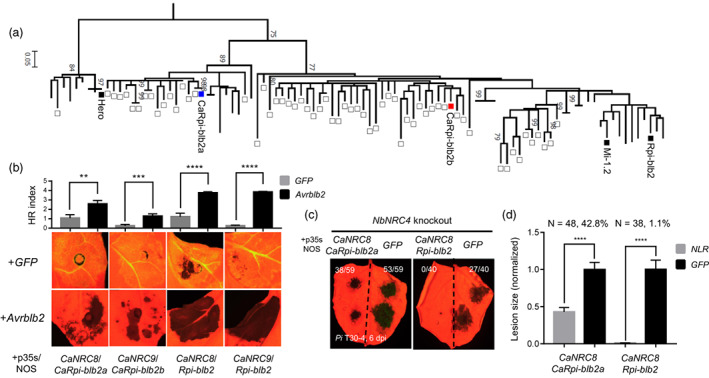
Pepper NLR pair (*CaNRC8/CaRpi‐blb2a*) trigger HR against Avrblb2 and resistance to *P. infestans* in *N. benthamiana*. (a) Phylogeny of CNL‐G1 NLRs (Rpi‐blb2 clade) of four Solanaceae plants (tomato, potato, pepper, and *N. benthamiana*) showing reference CNL‐G1 NLRs (Rpi‐blb2, Mi‐1.2, and Hero, black boxes), pepper NLRs (white boxes), CaRpi‐blb2a (blue box), and CaRpi‐blb2b (red box). (b) *CaNRC8/CaRpi‐blb2a* or *Rpi‐blb2*, *CaNRC9a/CaRpi‐blb2b* or *Rpi‐blb2* pairs triggered more intense HR‐like cell death when co‐expressed with Avrblb2 than with GFP (negative control). HR intensity of each combination was calculated by averaging the HR index of 12 infiltrated spots. Statistical significance was analysed using the *t*‐test (error bars represent SEM from 12 replicates; **P < 0.05; ***P < 0.005; ****P < 0. 0001). (c) Expression of *CaNRC8/CaRpi‐blb2a* conferred resistance against *Pi* T30‐4 compared to *GFP*‐expression in the other half of the same leaves in *NbNRC4*‐knockout *N. benthamiana*. The number of growing lesions/total inoculation sites is indicated on the upper right of each image. (d) Average lesion size of *Pi* T30‐4. Statistical significance was analysed using the *t*‐test (error bars indicate SEM; ****P < 0.0001).

Our functional validation of CaNRCs prompted us to revisit the screening to identify pepper NLRs that respond to Avrblb2s. We hypothesized that pepper NLRs belonging to the NRC‐S clade, especially the CNL‐G1 group that clustered with Rpi‐blb2 homologues (Seo *et al*., 2016), require pepper‐specific NRCs to recognize and trigger immune responses against Avrblb2 and *P. infestans*. Thus, 62 CNL‐G1 NLRs of pepper were co‐expressed with *Avrblb2* and *CaNRC8* or *CaNRC9a* in *N. benthamiana* (Figures 4a, S12; Table S3). For the efficient co‐expression, *Avrblb2* and *CaNRC8* or *9a* were cloned into dual gene expression cassette (Figure S13). As a result, two NLRs, *CaRpi‐blb2a* (Ca05g17760) and *CaRpi‐blb2b* (Ca00g87530), induced HR against *Avrblb2* only when co‐expressed with *CaNRC8* and *CaNRC9a*, respectively (Figures 4b, S14). Next, we tested whether the expression of *CaNRC8/CaRpi‐blb2a* or *CaNRC9a/CaRpi‐blb2b* pairs conferred resistance to *P. infestans*. Both NLR pairs were transiently co‐expressed on *NbNRC4*‐knockout *N. benthamiana* and inoculated with *P. infestans* (*Pi* T30‐4). The *CaNRC8/Rpi‐blb2a* pair significantly reduced lesion size of *Pi* T30‐4 and expression level of *P. infestans* actin compared to the control side of leaf expressing *GFP*, *CaNRC8*, or *CaRpi‐blb2a* (Figures 4c,d, S7, S15).

These combined results show that we identified pepper NLR recognizing Avrblb2 using a reverse genetics approach with *N. benthamiana* as a surrogate system, similar to the cases for Avr2, Avrblb1, and Avrvnt1. These observations support that pepper possesses multiple NLRs that retain similar recognition specificities with its *Solanum* homologues, thereby enabling pepper to recognize non‐adapted *P. infestans*. Our results also indicate that signalling compatibility between helper‐sensor NLRs should be carefully considered when transferring NLRs between distantly related plants like pepper and *N. benthamiana*.

### CaRpi‐blb2a is a functional homologue of Rpi‐blb2 but evades suppression by *P. infestans* effector PITG_15 278

The newly identified pepper NLRs are functional homologues (recognizing the same effectors) of previously reported NLRs derived from wild *Solanum* species, such as R2, Rpi‐blb1, Rpi‐blb2, and Rpi‐vnt1 (Figures 2a, 4a, S3, S12). Most *Solanum* NLRs, including Rpi‐blb1 and Rpi‐blb2, have been repeatedly overcome by virulent *P. infestans* isolates. Therefore, we tested whether functional homologues of pepper conferred more stable resistance against immune suppression by *P. infestans*.

**Figure 5:**
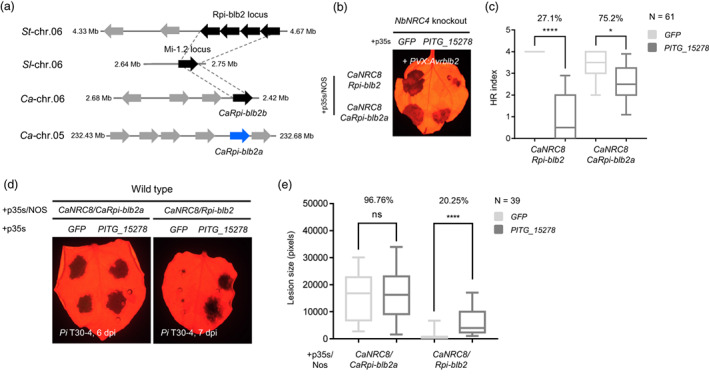
The pepper‐specific NLR pair *CaNRC8/CaRpi‐blb2a*‐mediated HR and resistance and were more tolerant to suppression by the PITG_15 278 effector. (a) Microsynteny of Rpi‐blb2 loci in potato (St), tomato (Sl), and pepper (Ca). Rpi‐blb2 locus and its corresponding orthologs of pepper (CaRpi‐blb2b loci) and tomato (Mi‐1.2 loci) are linked as dotted grey lines. NB‐ARC coding genes are presented as grey arrows, and Rpi‐blb2 orthologs are marked as black arrows. (b) *CaRpi‐blb2a*‐mediated HR cell death against Avrblb2 was more tolerant to PITG‐15278‐mediated suppression compared to *CaNRC8/Rpi‐blb2*. Photographs were taken at 4 dpi. (c) Average HR intensities of the presented combinations. Statistical significance of variance was analysed using the F‐test (****P < 0.0001; *P < 0.05). (d) *CaNRC8/CaRpi‐blb2a*‐mediated resistance was more tolerant to suppression by PITG‐15278‐expression compared to *CaNRC8/Rpi‐blb2*. (e) Average lesion sizes are presented in a box plot. Statistical significance was analysed using the unpaired *t*‐test (ns, nonsignificant; ****P < 0.0001).

The *P. infestans* effector PITG_15 278 was reported to suppress *Rpi‐blb2*‐mediated cell death (Derevnina *et al*., 2021). We co‐expressed PITG_15 278 with *CaNRC8/Rpi‐blb2* or *CaRpi‐blb2a* in the NbNRC4‐knockout plant. The results showed that *CaNRC8/CaRpi‐blb2a*‐mediated HR was significantly less compromised by the expression of PITG_15 278 compared to that of *CaNRC8*/*Rpi‐blb2* (Figure 5b,c). *CaNRC8/CaRpi‐blb2a*–mediated resistance against *P. infestans* also was more tolerant to PITG_15 278 compared to that of *CaNRC8/Rpi‐blb2* in *N. benthamiana* (Figure 5d,e).

Although *CaRpi‐blb2a* is a homologue of *Rpi‐blb2* and classified as CNL‐G1, *CaRpi‐blb2a* was distinctly clustered with pepper NLRs and clearly separated from the Rpi‐blb2 clade in the phylogenetic tree (Figure 4a). Moreover, *CaRpi‐blb2a* was located on chromosome 5, distinct from the clusters of G1‐NLRs located on chromosome 6 (Rpi‐blb2 loci) (Figure 5a). Furthermore, CaRpi‐blb2a exhibited similar, but distinguished recognition specificity compared to Rpi‐blb2 (Figure S16). These results indicate that pepper has evolved functionally homologous but distinct NLRs, compared to its *Solanum* homologue Rpi‐blb2. This difference may contribute to the tolerance of *CaRpi‐blb2a* against PITG_15 278, which evolved to suppress the immune system of its host plants.

Similarly, the IPIO‐4 variant of Avrblb1 was reported to suppress Rpi‐blb1‐mediated HR against Avrblb1 by directly binding to Rpi‐blb1 (Champouret *et al*., 2009; Chen *et al*., 2012; Zhao and Song, 2021). However, *CaRpi‐blb1b* exhibited a similar level of cell death as *Rpi‐blb1* when co‐expressed with IPIO‐4 (Figure S17), which differed from the case of *Rpi‐blb2* and *CaRpi‐blb2a*. These results indicate that pepper possesses multiple homologous NLRs that retain similar effector recognition specificities with its *Solanum* homologues, and some of these pepper NLRs could be more tolerant to immune suppression by the non‐adapted pathogen *P. infestans*.

## Discussion

### Nonhost plants possess multiple NLRs that recognize non‐adapted pathogen effectors

**Figure 6:**
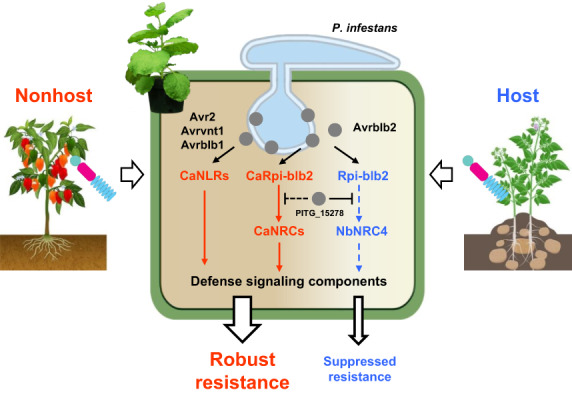
Mechanistic model of NLR network–mediated pepper NHR against *P. infestans*. Pepper is diverged from host plants of *P. infestans* in the Solanaceae (potato and tomato) 20 million years ago; however, pepper still possess multiple NLRs that recognize *P. infestans* effectors. Some of these NLRs could be more tolerant to immune suppression mechanisms of *P. infestans* compared to functional homologues of host plants. This NLR network may contribute to the robust NHR of pepper against the non‐adapted *P. infestans* and could be exploited to confer durable resistance in susceptible crops such as potato and tomato.

This study showed that the NLR network of a nonhost plant could be exploited to confer resistance against a non‐adapted pathogen. Nonhost pepper possesses multiple NLRs homologous to *Solanum* NLRs and recognized the same effectors of the non‐adapted pathogen *P. infestans* (Figures 2, 4). Therefore, we propose a model in which pepper's NHR is the result of a stack of multiple R genes, some of which evade suppression by *P. infestans* effectors (Figure 6). Similar with our results, several NLRs that recognize the effectors of adapted pathogens have been reported to also recognize non‐adapted pathogens. For example, the barley NLR *mildew locus a* homologue (Mla8, RpS9) conferred resistance to adapted *Blumeria graminis* f. sp. *hordei* and non‐adapted *Puccinia striiformis* f. sp. *tritici* (Saur *et al*., 2022). The Rpi‐amr1/3 NLRs derived from *Solanum americanum* were reported to recognize *P. infestans* effectors and homologues derived from several related *Phytophthora* spp. (Lin *et al*., 2022; Witek *et al*., 2021). These results indicate that plants recognize non‐adapted pathogen effectors that are adapted to closely related plant species. We proposed that structural (directly recognized by similar NLRs) or functional (interacted with similar host targets guarded by NLRs) homologies conserved among pathogen effectors may enable plants to recognize effectors derived from adapted and non‐adapted pathogens (Oh and Choi, 2022).

Multiple homologous NLR/effector pairs conserved in taxonomically related plant/pathogen species likely enable cross‐recognition, and these NLRs may contribute to the underlying molecular mechanisms of NHR (Schulze‐Lefert and Panstruga, 2011). This hypothesis is consistent with the successful introduction of nonhost plant NLRs into closely related (intrafamily) plants, such as maize to rice (Zhao *et al*., 2004, 2005), Arabidopsis to mustard (Borhan *et al*., 2008, 2010), pigeon pea to soybean (Kawashima *et al*., 2016), and barley to wheat, respectively (Bettgenhaeuser *et al*., 2021). In addition, most of the previous studies only reported single functional NLRs from each system. By contrast, our approach identified multiple NLRs through homology‐based genome‐wide screening. We expect that implementing this approach in other pathosystems will enable the discovery of a vast array of NLRs recognizing non‐adapted pathogen effectors.

### Nonhost NLR‐mediated resistance could be more tolerant to immune suppression by a pathogen

CaRpi‐blb2a is homologues of Rpi‐blb2, and CaRpi‐blb2a is more tolerant to immune suppression by the *P. infestans* effectors PITG_15 278 compared to Rpi‐blb2 from *Solanum* species. These results suggest that NLRs derived from nonhost plants could be more tolerant to suppression by non‐adapted pathogens than the NLRs of host species to a given pathogen. This may result from the lack of adaptation of the pathogen to the defence components of nonhost plants after speciation from the common ancestor, while nonhost plants may retain recognition specificities against non‐adapted pathogens.

Similar to our results, several R genes have been reported as more tolerant to suppression by non‐adapted pathogens, whereas adapted pathogens are capable of suppressing given R genes. For example, wheat NLR Pm3 recognizes Avrpm3 of wheat‐adapted *Blumera graminis* f. sp. *tritici* (*Bgt*) and Avrpm3 homologues of *B. graminis* f. sp *secalis* (*Bgs*) and *dactylidis* (*Bgd*), which are non‐adapted to wheat. In this pathosystem, Svrpm3 of *Bgt* suppresses Pm3, whereas Svrpm3 homologues derived from *Bgs* and *Bgd* cannot suppress Pm3 (Bourras *et al*., 2019). Orthologous pathogen‐associated molecular pattern (PAMP) receptors Rphq2 and Rph22 are derived from cultivated and wild barley, respectively, and conferred more intensive resistance against its non‐adapted pathogens when transferred into experimentally susceptible barley lines (Wang *et al*., 2019).

We propose that the NLR networks of taxonomically related nonhost plants retain recognition specificities against non‐adapted pathogens and could be more tolerant to immune suppression mechanisms of the corresponding non‐adapted pathogens. Thus, nonhost Solanaceae plants such as pepper would provide an untapped resource of NLRs for developing *P. infestans* resistance, whereas NLRs derived from wild *Solanum* species are repeatedly overcome by pathogen variants (Figure 6).

### Understanding the structures and functions of lineage‐specific NLR network architectures is crucial for exploiting NLRs from nonhost plants

In general, NLRs cannot be genetically transferred by crossing between evolutionarily distant host and nonhost plant species. In these cases, heterologous expression is the only way to transfer NLRs from nonhost to host. We observed functional incompatibility of helper and sensor NLRs between pepper and *N. benthamiana* (Figures 3, 4). Therefore, understanding the NLR signalling network architecture could be crucial for effective transfer of functional NLR genes among evolutionarily distant plant species. For example, inter‐family transfer of pepper NLR BS2 (Tai *et al*., 1999) into cassava failed to confer resistance against *Xanthomonas axonopodis* (Díaz‐tatis *et al*., 2019), whereas transfer into BS2‐expressing tomato confers resistance to a similar pathogen, *Xanthomonas euvesicatoria* (Horvath *et al*., 2012). As both of these *Xanthomonas* pathogens contain AvrBs2 homologues, these results could be due to the lack of NRC in cassava or the incompatibility between cassava helper NLRs and the pepper sensor NLR, BS2. Similarly, each component of the broadly conserved EDS1‐SAG101‐NRG1 signalling modules is compatible between *N. benthamiana* and tomato but is not compatible with Brassicaceae *Arabidopsis* (Lapin *et al*., 2019). These results suggest that understanding the compatibilities between the NLR signalling components and the co‐transfer of functional combinations is crucial for conferring disease resistance when exploiting NLRs derived from evolutionarily distant plant species.

As we presented that *NbNRC4* is not working with *CaRpi‐blb2a* while *CaNRC8/9* is functionally compatible with Rpi‐blb2, more lineage‐specific helper NLRs with specific sensor‐helper compatibility could be identified. Indeed, most of Solanaceae plants still retain its own NRC4‐like clade (Figure S8) which could be the result of divergent evolution. To date, most of studies are focused on functionally and structurally conserved *NRC1/2/3/4* homologues. We anticipate that further investigation on the sensor‐helper compatibilities of these lineage‐specific helper NLRs of Solanaceae plants would be needed as more sensor NLRs are identified in various Solanaceae plants.

A number of NLRs have been identified and the majority depend on helper NLRs such as NRCs, NRG1, and ADR1 for their function (Kamoun *et al*., 2018; Ngou *et al*., 2022). Thus, helper NLRs could be ideal targets for pathogens to efficiently overcome multiple NLR‐mediated immune responses of plants (Li *et al*., 2021). Divergent pathogens possess multiple effectors that function as suppressors of helper NLRs, such as Avrcab1b of *P. infestans* and SS17 of nematodes that suppresses NbNRC2/3 (Derevnina *et al*., 2021). In this context, we could assume that helper NLRs derived from nonhost plants would be more tolerant to suppression by non‐adapted pathogens as we observed that sensor NLRs derived from pepper were more tolerant to suppression by *P. infestans* compared to *Solanum*‐derived homologues (Figure 5). This indicates that co‐transfer of functional homologues of helper NLRs derived from nonhost plants together with the multiple sensor NLRs would enhance plant defence against adapted pathogens that suppress host helper NLRs.

In conclusion, our study provide evidence that multiple NLRs mediate recognition of non‐adapted pathogen effectors when the nonhost plant (pepper) is closely related (intrafamily) to the host plant (potato). This result is consistent with previous hypothesis about the relationship between evolutionary distances of nonhost/host plants and the molecular mechanisms of receptor‐mediated nonhost resistance (Oh and Choi, 2022; Schulze‐Lefert and Panstruga, 2011). Moreover, some of these nonhost plant–derived NLRs could be more tolerant to immune suppression by pathogens, whereas NLRs derived from wild *Solanum* species, which are closely related to the original host (potato, tomato) of *P. infestans*, have been repeatedly overcome by *P. infestans*. Therefore, further identification and understanding of NLRs derived from distantly related nonhost plants would provide a promising strategy for developing durable resistant crops against devastating pathogens such as *P. infestans*. We also expect nonhost plants could be untapped resources for future development of disease resistant crops through biotechnology.

## Materials and methods

### Plant materials and growth conditions

Pepper (*C. annuum* cv. CM334 and ECW30R), tobacco (*N. benthamiana* wild type, NLR transgenic, *NbNRC4* CRISPR‐knockout lines SK185.1.2.1 and SK185.9.1.3), and potato (*S. tuberosum* cv. Desiree) plants were grown in a controlled chamber at 24–26°C and 40–60% relative humidity with a 16‐h light/8‐h dark cycle.

### *Phytophthora infestans* materials and inoculation methods

*Phytophthora infestans* isolates (T30‐4 and NL07434) were grown on rye agar plate media in a dark chamber at 17–19°C for 7–9 days. Zoospores were harvested from flooded (with 6–8 mL of TDW) plates after incubating at 4°C for 1 h. Whole plants were sprayed with zoospore solution (5.0 × 10^4^ zoospores/mL) and placed in a growth chamber under the same conditions as described above for plant growth. For the detached leaf assay, the abaxial side of the detached leaves of pepper, potato, and *N. benthamiana* was inoculated with 11 μL of zoospore solution (1.0 × 10^5^ zoospores/mL) and placed in SPL® rectangular plates with wet tissue paper to maintain 100% relative humidity. Inoculated leaves were incubated at 21–23°C with 16‐h light/8‐h dark cycle until further examination. Detailed information for each experiment is provided in the Supplementary Methods.

### Leaf infiltration

Agrobacterium strain GV3103 containing each construct was cultured in YEP medium for 1 day. Pellets were collected by centrifugation (10 min, 1900 *g*), resuspended into Agroinfiltration buffer (10 mm MES, 10 mm MgCl_2_, 150 μm acetosyringone, pH 5.6). Detailed information for each experiment is provided in Supplementary Methods S1.

### PVX virion infection

Fifty‐seven *P. infestans* RXLR effectors were chemically synthesized, cloned into the PVX vector (pICH31160), and transformed into Agrobacterium strain GV3101. Agrobacterium was infiltrated into first and second true leaves of 3‐week‐old *N. benthamiana* to propagate PVX virions. The uninoculated systemic leaves were harvested, freeze dryed and ground into fine dust at 8–10 days post inoculation (dpi) to prepare virion inoculum. The inoculum powder was suspended in 0.05 m potassium phosphate buffer (pH 7.4), mixed with 400 mesh carborundum, and rubbed on 4‐week‐old CM334 and ECW30R pepper leaves. Inoculated leaves were detached at 7 dpi and destained with 100% ethanol for 2 days to visualize HR phenotypes.

### Cloning of pepper NLRs

NLRs containing at least 160 amino acids of the NB‐ARC domain and three major (P‐loop, kinase, GLPL, or MDHV) and minor (RNBS‐A, RNBS‐B, RNBS‐C, RNBS‐D) motifs were defined as full NLRs. Using these criteria, 426 pepper NLRs (Table S3) were predicted based on the CM334 pepper genome version 1.55 coding sequence (CDS) database as described previously (Seo *et al*., 2016). The gene boundary and structure of each NLR were reconfirmed by performing BLASTn using a default database (Nucleotide collection nr/nt) and the Softberry Fgenesh tool. Then, 0.5–1 kb flanking regions of predicted start/end codons of each NLR were amplified using Primestar GXL (TAKARA®) enzyme from genomic DNA of CM334 pepper. Each amplicon was cloned into the pCAMBIA2300‐LIC vector (p35s) using the ligation‐independent cloning (LIC) method and the cloned sequences were validated by Sanger sequencing (Aslanidis and De Jong, 1990; Oh *et al*., 2010).

### Phylogenetic analysis of NLRs

The NLR parser (Steuernagel *et al*., 2015) identified NLR sequences from four Solanaceae plants using public CDS databases including pepper version 2.0, tomato ITAG4.0, potato DM_v3.4, and *N. benthamiana* version 1.0.1. Known R gene sequences (a total of 33) gathered from the plant resistance gene database (PRGDB) and other published references (Table S5) were added to the data set as reference genes for clade identification. Two potato and one tomato NRC3 protein sequences were manually added to the data set because these sequences were missing in the public annotation (Wu *et al*., 2017). Only complete forms of NLRs predicted from the NLR parser were used for analysis, but cloned pepper NLRs (which are filtered out by the NLR parser) were added to the data set. The whole NLRs were aligned using the MUSCLE (default setting) algorithm, and a maximum‐likelihood phylogenetic tree was constructed using MEGA7 (Kumar et al., 2016) with 500× bootstraps and 0.8–0.9 gap deletion parameters for each analysis. NLR clades were defined based on previously published NLR groups as described in Seo *et al*. (2016) using reference genes and BLAST searches. The subtrees of CNL‐G1 (containing Rpi‐blb2, Mi‐1.2, and Hero), CNL‐G5 (R2, Rpi‐blb3), CNL‐G7 (Rpi‐blb1), CNL‐G8 (NRC clade), and CNL‐G11 (Rpi‐vnt1, Tm2) were extracted from the whole tree and presented in each figure.

### Virus‐induced gene silencing

To silence *NbNRC2/3/4* with a single construct, fragments of *NbNRC2* (1–285), *NbNRC3* (1–334), and *NbNRC4* (1–272) transcripts, with regions covering all functional homologues of NRC2/3/4 (Wu *et al*., 2017), were linked through overlap PCR and cloned into tobacco rattle virus RNA2 vector (pTRV2‐LIC). Agrobacterium‐containing pTRV1 and pTRV2:*NbNRC2/3/4* were suspended in Agroinfiltration buffer, adjusted to OD_600_ = 0.4, and mixed in a 1:1 ratio. The first true leaves of 2‐week‐old *N. benthamiana* were infiltrated with Agrobacterium and used for silencing confirmation and functional analyses at 2–3 weeks after TRV inoculation. NRC silencing was confirmed by performing quantitative RT‐PCR using the SYBR green master mix (Thermo Fisher Scientific®: Waltham, MA USA).

### Microsynteny analyses

The MCScan (https://github.com/tanghaibao/jcvi/wiki/MCscan) program was used for microsynteny analysis of pepper, potato, and tomato genomes (Tang *et al*., 2008). Coding DNA sequences from pepper (version 2.0), tomato (ITAG 4.0), and potato (PGSC version 3.4) genomes were extracted for analysis. General feature format (gff) files for physical locations were generated and used for MCScan analysis using default settings except for ‐‐cscore 0.99 and ‐‐iter 1 parameter. Corresponding synteny blocks of the Rpi‐blb2 locus (putative Rpi‐blb2, Mi‐1.2, and CaRpi‐blb2b loci of potato, tomato, and pepper, respectively) were simplified and presented in figures with the CaRpi‐blb2a locus.

## Conflict of interest

These authors declare no competing interests.

## Author contributions

S.O., H.L., and D.C. designed the research. S.O., S. Kim, H.P., H.L., M.K., M.S., and H.K. performed research. S.O., M.K., and C.W. analysed the data. S.O., S.K., and D.C. wrote the paper.

## Supporting information


**Figure S1** PVX virion‐mediated gene expression using PVX‐GFP on pepper accessions (CM334 and ECW) and *Nicotiana benthamiana*.
**Figure S2** Effector screening result via agrobacterium‐mediated transient expression of effectors on CM334 pepper (error bars indicate SEM from 4 replicates for negative cases and at least 18 for positive cases).
**Figure S3** Detailed (named) phylogenetic tree of screened CaNLRs (G5, G7, G11) and potato NLRs.
**Figure S4** Expression of pKW:Avrvnt1 (PITG_16294) induced cell death itself in *N. benthamiana* while p35s:Avrvnt1 was not.
**Figure S5** Average lesion size data obtained from five independent experiments were presented with 10‐90 percentile box plot.
**Figure S6** Lesion size of *Phytophthora infestans* NL07434 is correlated with *Pi* biomass in transgenic *Nicotiana benthamiana* with different level of *Rpi‐blb2* expression.
**Figure S7** Decreased lesion size of *Phytophthora infestans* is correlated with expression level of *P. infestans* actin in *CaNLR‐*expressed *Nicotiana benthamiana* leaves.
**Figure S8** Maximum‐likelihood (bootstraps = 500) phylogenetic tree of CNL‐G8 NLRs of four Solanaceae species (tomato, potato, pepper, and *N. benthamiana*).
**Figure S9** (A) Amino acid sequences of CaNRC1/2/8/9 and NbNRC2/3/4 were aligned with ClustaW and visualized with BoxShade tool. (B) Reads per kilo base of transcript per million mapped reads (RPKM) of 755 pepper NLRs were obtained from transcriptome data of pepper leaves infected with *P. infestans* (Kim *et al*., 2018), and presented as a dot plot.
**Figure S10** Virus‐induced gene silencing (VIGS) of *NbNRC2/3/4* significantly compromised known NRC‐dependent sensor (NRC‐S) NLRs (R1, R8, and Rpi‐blb2)‐mediated HR against each corresponding effector.
**Figure S11** *CaNRC4a/b* are not expressed as intact from (containing CC‐NB‐ARC‐LRR domains) in CM334 pepper.
**Figure S12** Phylogeny of Solanaceae CNL‐G1 NLRs. Maximum‐likelihood phylogenetic tree (bootstraps = 500) of CNL‐G1 NLRs of 4 Solanaceae plants (tomato, potato, pepper, and *N. benthamiana*).
**Figure S13** Functional validation of dual‐expression cassette for co‐expression assay (pD35/NOS).
**Figure S14** Amino acid sequences of CaRpi‐blb2a and CaRpi‐blb2b.
**Figure S15** Expression of *CaNRC8/CaRpi‐blb2a* also significantly reduced average lesion size of *P. infestans* when compared to *CaRpi‐blb2* or *CaNRC8*‐expressed in half of the same leaves of *N. benthamiana*.
**Figure S16** Recognition spectrum of *Rpi‐blb2*, *CaRpi‐blb2a*, and *CaRpi‐blb2b*.
**Figure S17** *CaRpi‐blb1b*‐mediated HR cell death against Avrblb1 is similarly suppressed by IPIO4 compared to *Rpi‐blb1*.

**Table S1** Cell death screening results of 57 *P. infestans* RxLR effectors using PVX‐inoculation method.
**Table S2** Classification of Cloned 436 pepper NLRs.
**Table S3** Whole cloned CaNLRs sequences.
**Table S4** Lists of pepper CNL‐G5, G7, and G11 for co‐expression assay with Avr2, Avrblb1, and Avrvnt1, respectively.
**Table S5** Lists of known R genes used for phylogenetic analyses.
**Table S6** Lists of primers used to cloning NRCs.
**Table S7** Lists of primers used to cloning known R genes and AVR effectors.
**Table S8** Lists of primers used for qRT‐PCR.
**Table S9** Converting Id of pepper CNL‐G1 NLRs from 1.55v to 2.0v.

**Appendix S1** Materials and Methods.

## Data Availability

All data discussed in this study can be found in the manuscript and Supplementary Materials.
