# Supplementary material for: Nucleotide‐binding leucine‐rich repeat network underlies nonhost resistance of pepper against the Irish potato famine pathogen Phytophthora infestans
Source: Plant Biotechnol J. 2023 Mar 13;21(7):1361–72. doi: 10.1111/pbi.14039 (PMC10281606; doi:10.1111/pbi.14039)
Supplement: Supplementary file 1 — Figure S1 PVX virion‐mediated gene expression using PVX‐GFP on pepper accessions (CM334 and ECW) and Nicotiana benthamiana. Figure S2 Effector screening result via agrobacterium‐mediated transient expression of effectors on CM334 pepper (error bars indicate SEM from 4 replicates for negative cases and at least 18 for positive cases). Figure S3 Detailed (named) phylogenetic tree of screened CaNLRs (G5, G7, G11) and potato NLRs. Figure S4 Expression of pKW:Avrvnt1 (PITG_16294) induced cell death itself in N. benthamiana while p35s:Avrvnt1 was not. Figure S5 Average lesion size data obtained from five independent experiments were presented with 10‐90 percentile box plot. Figure S6 Lesion size of Phytophthora infestans NL07434 is correlated with Pi biomass in transgenic Nicotiana benthamiana with different level of Rpi‐blb2 expression. Figure S7 Decreased lesion size of Phytophthora infestans is correlated with expression level of P. infestans actin in CaNLR‐expressed Nicotiana benthamiana leaves. Figure S8 Maximum‐likelihood (bootstraps = 500) phylogenetic tree of CNL‐G8 NLRs of four Solanaceae species (tomato, potato, pepper, and N. benthamiana). Figure S9 (A) Amino acid sequences of CaNRC1/2/8/9 and NbNRC2/3/4 were aligned with ClustaW and visualized with BoxShade tool. (B) Reads per kilo base of transcript per million mapped reads (RPKM) of 755 pepper NLRs were obtained from transcriptome data of pepper leaves infected with P. infestans (Kim et al., 2018), and presented as a dot plot. Figure S10 Virus‐induced gene silencing (VIGS) of NbNRC2/3/4 significantly compromised known NRC‐dependent sensor (NRC‐S) NLRs (R1, R8, and Rpi‐blb2)‐mediated HR against each corresponding effector. Figure S11 CaNRC4a/b are not expressed as intact from (containing CC‐NB‐ARC‐LRR domains) in CM334 pepper. Figure S12 Phylogeny of Solanaceae CNL‐G1 NLRs. Maximum‐likelihood phylogenetic tree (bootstraps = 500) of CNL‐G1 NLRs of 4 Solanaceae plants (tomato, potato, pepper, and N. benthamiana [file PBI-21-1361-s003.pptx]

## Slide 1
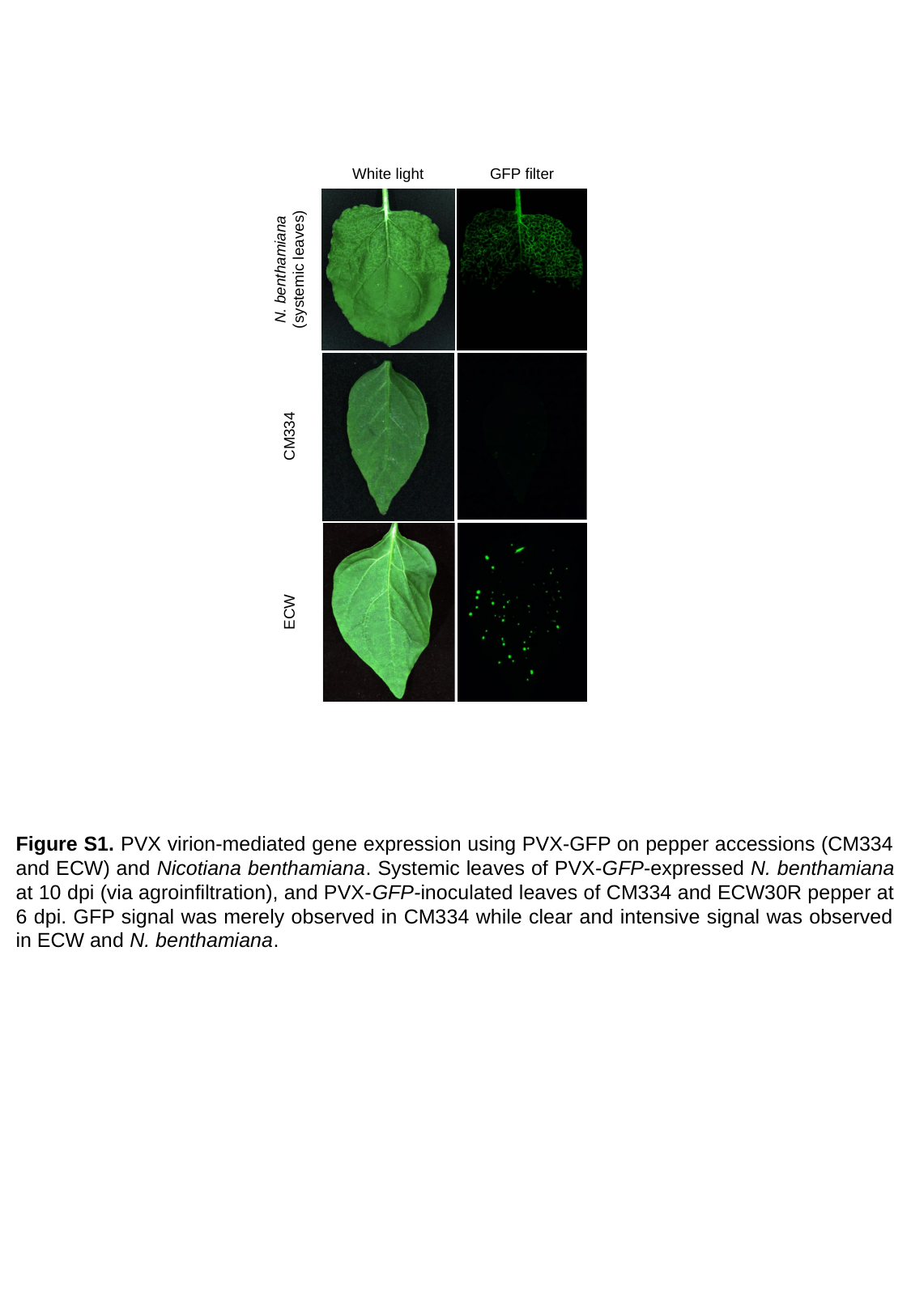

GFP filter
White light
N. benthamiana
(systemic leaves)
CM334
ECW
Figure S1. PVX virion-mediated gene expression using PVX-GFP on pepper accessions (CM334 and ECW) and Nicotiana benthamiana. Systemic leaves of PVX-GFP-expressed N. benthamiana at 10 dpi (via agroinfiltration), and PVX-GFP-inoculated leaves of CM334 and ECW30R pepper at 6 dpi. GFP signal was merely observed in CM334 while clear and intensive signal was observed in ECW and N. benthamiana.

## Slide 2
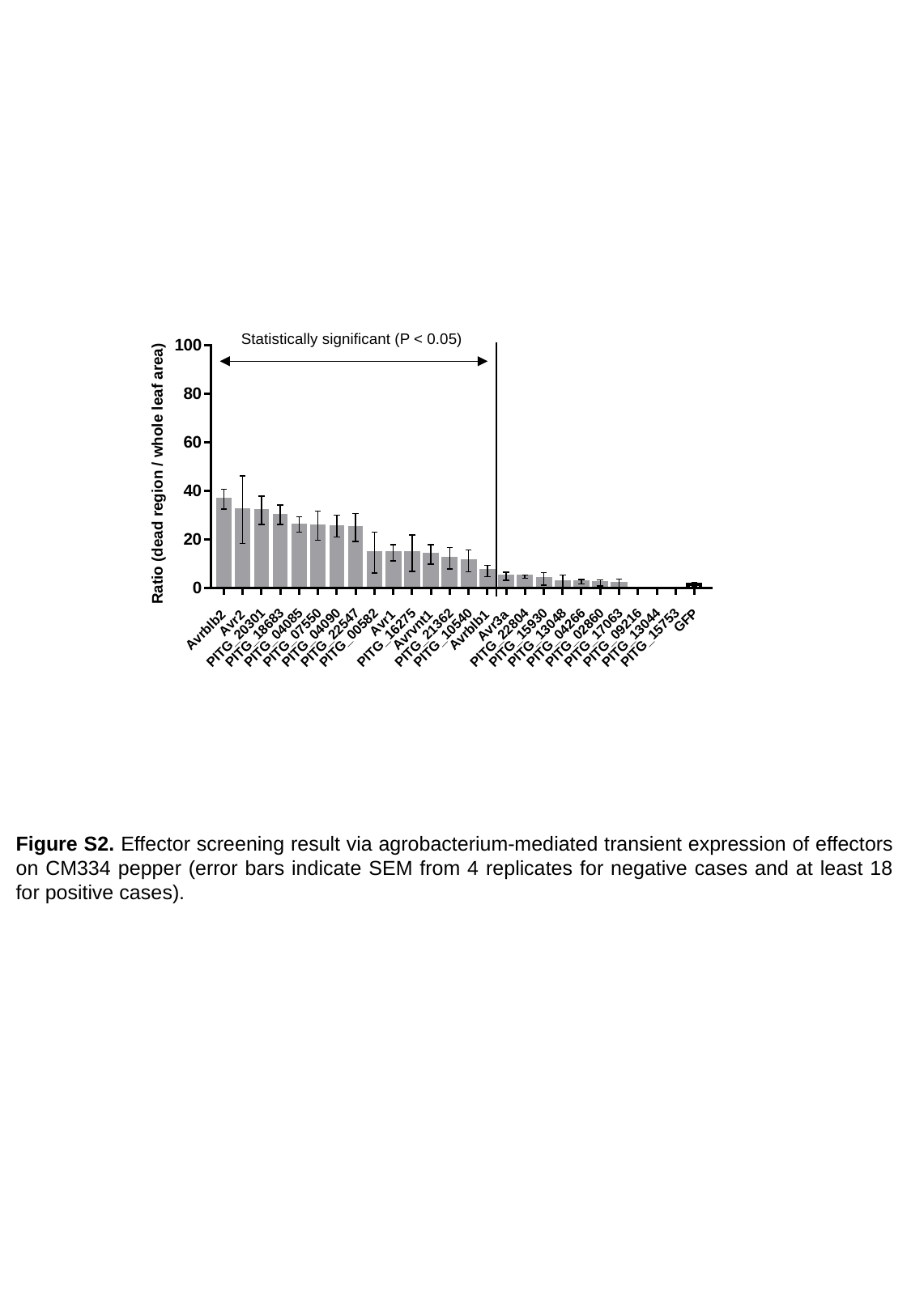

Statistically significant (P < 0.05)
Ratio (dead region / whole leaf area)
Figure S2. Effector screening result via agrobacterium-mediated transient expression of effectors on CM334 pepper (error bars indicate SEM from 4 replicates for negative cases and at least 18 for positive cases).

## Slide 3
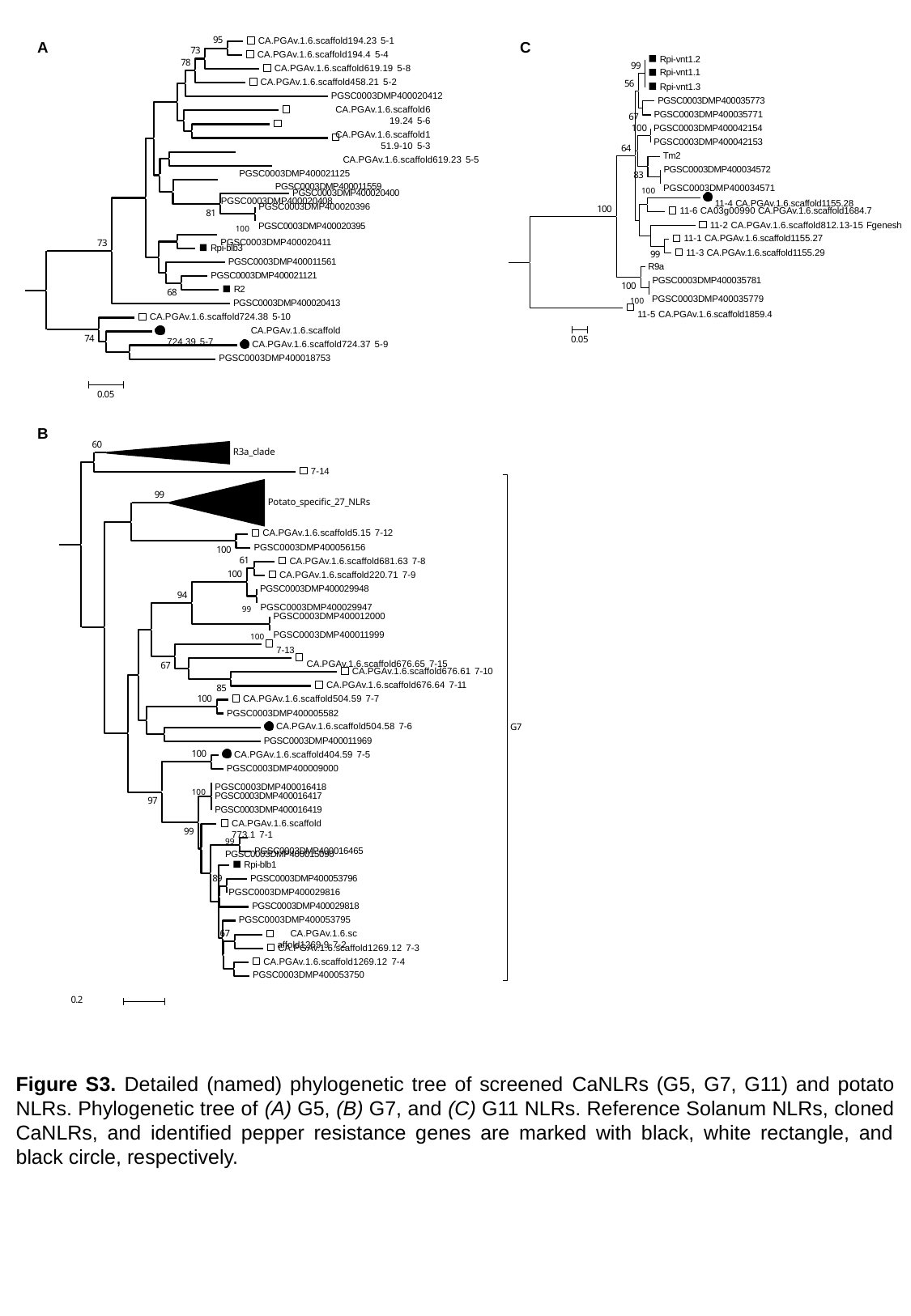

C
A
95
CA.PGAv.1.6.scaffold194.23 5-1
73
78
CA.PGAv.1.6.scaffold194.4 5-4
CA.PGAv.1.6.scaffold619.19 5-8
CA.PGAv.1.6.scaffold458.21 5-2
PGSC0003DMP400020412
CA.PGAv.1.6.scaffold619.24 5-6
CA.PGAv.1.6.scaffold151.9-10 5-3
CA.PGAv.1.6.scaffold619.23 5-5 PGSC0003DMP400021125
PGSC0003DMP400011559 PGSC0003DMP400020408
PGSC0003DMP400020400 PGSC0003DMP400020396
81
100 PGSC0003DMP400020395 PGSC0003DMP400020411
73
Rpi-blb3
PGSC0003DMP400011561
PGSC0003DMP400021121 R2
68
PGSC0003DMP400020413 CA.PGAv.1.6.scaffold724.38 5-10
CA.PGAv.1.6.scaffold724.39 5-7
74
CA.PGAv.1.6.scaffold724.37 5-9 PGSC0003DMP400018753
0.05
Rpi-vnt1.2 Rpi-vnt1.1
99
56
Rpi-vnt1.3 PGSC0003DMP400035773
PGSC0003DMP400035771
67
100 PGSC0003DMP400042154
PGSC0003DMP400042153
64
Tm2 PGSC0003DMP400034572
83
100 PGSC0003DMP400034571
11-4 CA.PGAv.1.6.scaffold1155.28
100
11-6 CA03g00990 CA.PGAv.1.6.scaffold1684.7
11-2 CA.PGAv.1.6.scaffold812.13-15 Fgenesh
11-1 CA.PGAv.1.6.scaffold1155.27
11-3 CA.PGAv.1.6.scaffold1155.29
99
R9a
PGSC0003DMP400035781
100
100 PGSC0003DMP400035779
11-5 CA.PGAv.1.6.scaffold1859.4
0.05
B
60
R3a_clade
7-14
99
Potato_specific_27_NLRs
CA.PGAv.1.6.scaffold5.15 7-12 PGSC0003DMP400056156
100
61
CA.PGAv.1.6.scaffold681.63 7-8
100
CA.PGAv.1.6.scaffold220.71 7-9
PGSC0003DMP400029948
94
99 PGSC0003DMP400029947
PGSC0003DMP400012000
100 PGSC0003DMP400011999
7-13
CA.PGAv.1.6.scaffold676.65 7-15
67
CA.PGAv.1.6.scaffold676.61 7-10
CA.PGAv.1.6.scaffold676.64 7-11
85
100
CA.PGAv.1.6.scaffold504.59 7-7
PGSC0003DMP400005582
CA.PGAv.1.6.scaffold504.58 7-6
G7
PGSC0003DMP400011969
CA.PGAv.1.6.scaffold404.59 7-5 PGSC0003DMP400009000
100
100 PGSC0003DMP400016418
PGSC0003DMP400016417 PGSC0003DMP400016419
CA.PGAv.1.6.scaffold773.1 7-1
97
99
99 PGSC0003DMP400015090
PGSC0003DMP400016465
Rpi-blb1 PGSC0003DMP400053796
PGSC0003DMP400029816
89
PGSC0003DMP400029818 PGSC0003DMP400053795
CA.PGAv.1.6.scaffold1269.9 7-2
67
CA.PGAv.1.6.scaffold1269.12 7-3
CA.PGAv.1.6.scaffold1269.12 7-4 PGSC0003DMP400053750
0.2
Figure S3. Detailed (named) phylogenetic tree of screened CaNLRs (G5, G7, G11) and potato NLRs. Phylogenetic tree of (A) G5, (B) G7, and (C) G11 NLRs. Reference Solanum NLRs, cloned CaNLRs, and identified pepper resistance genes are marked with black, white rectangle, and black circle, respectively.

## Slide 4
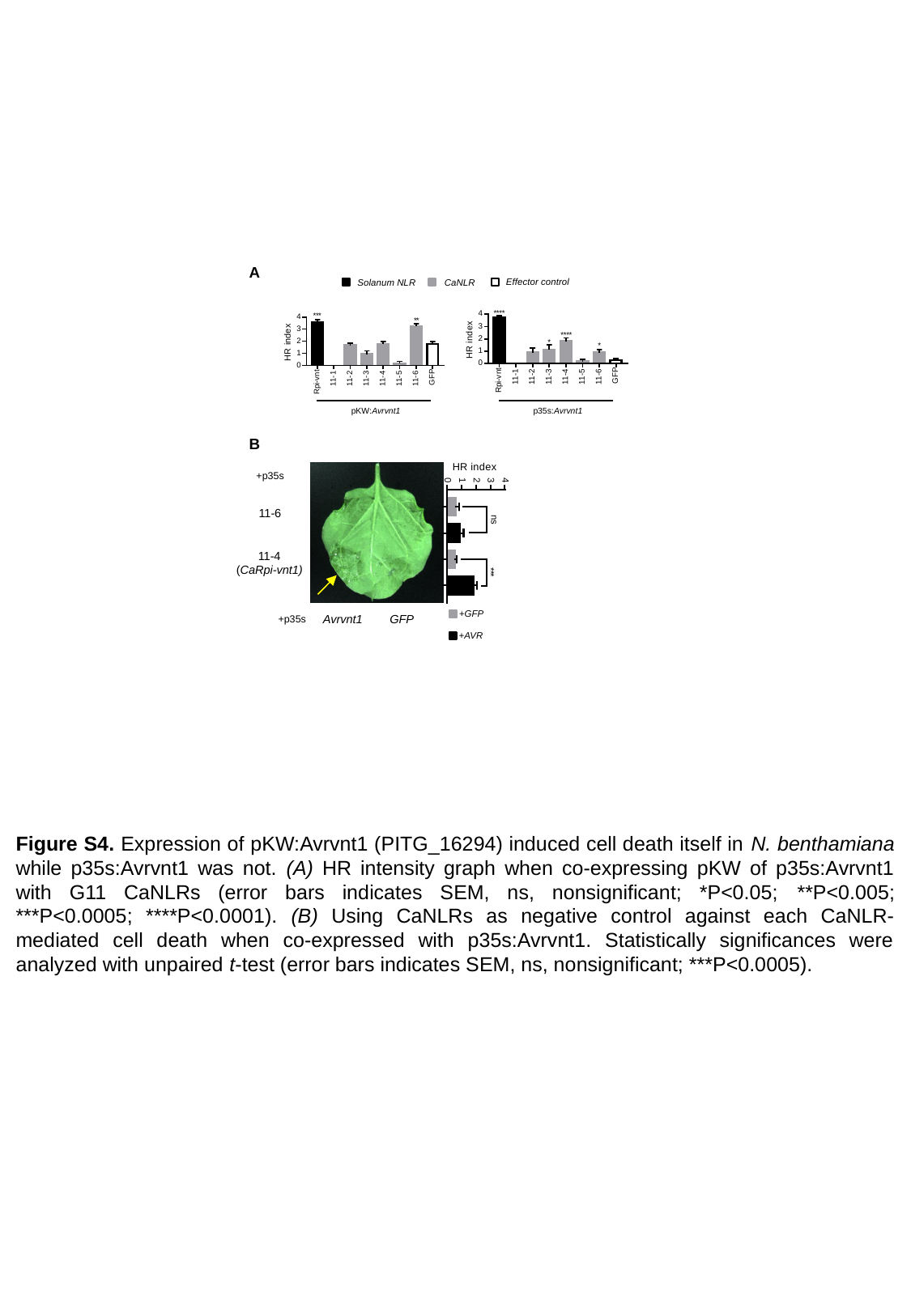

A
Effector control
CaNLR
Solanum NLR
pKW:Avrvnt1
p35s:Avrvnt1
B
+p35s
11-6
11-4
(CaRpi-vnt1)
+GFP
+AVR
Avrvnt1
GFP
+p35s
Figure S4. Expression of pKW:Avrvnt1 (PITG_16294) induced cell death itself in N. benthamiana while p35s:Avrvnt1 was not. (A) HR intensity graph when co-expressing pKW of p35s:Avrvnt1 with G11 CaNLRs (error bars indicates SEM, ns, nonsignificant; *P<0.05; **P<0.005; ***P<0.0005; ****P<0.0001). (B) Using CaNLRs as negative control against each CaNLR-mediated cell death when co-expressed with p35s:Avrvnt1. Statistically significances were analyzed with unpaired t-test (error bars indicates SEM, ns, nonsignificant; ***P<0.0005).

## Slide 5
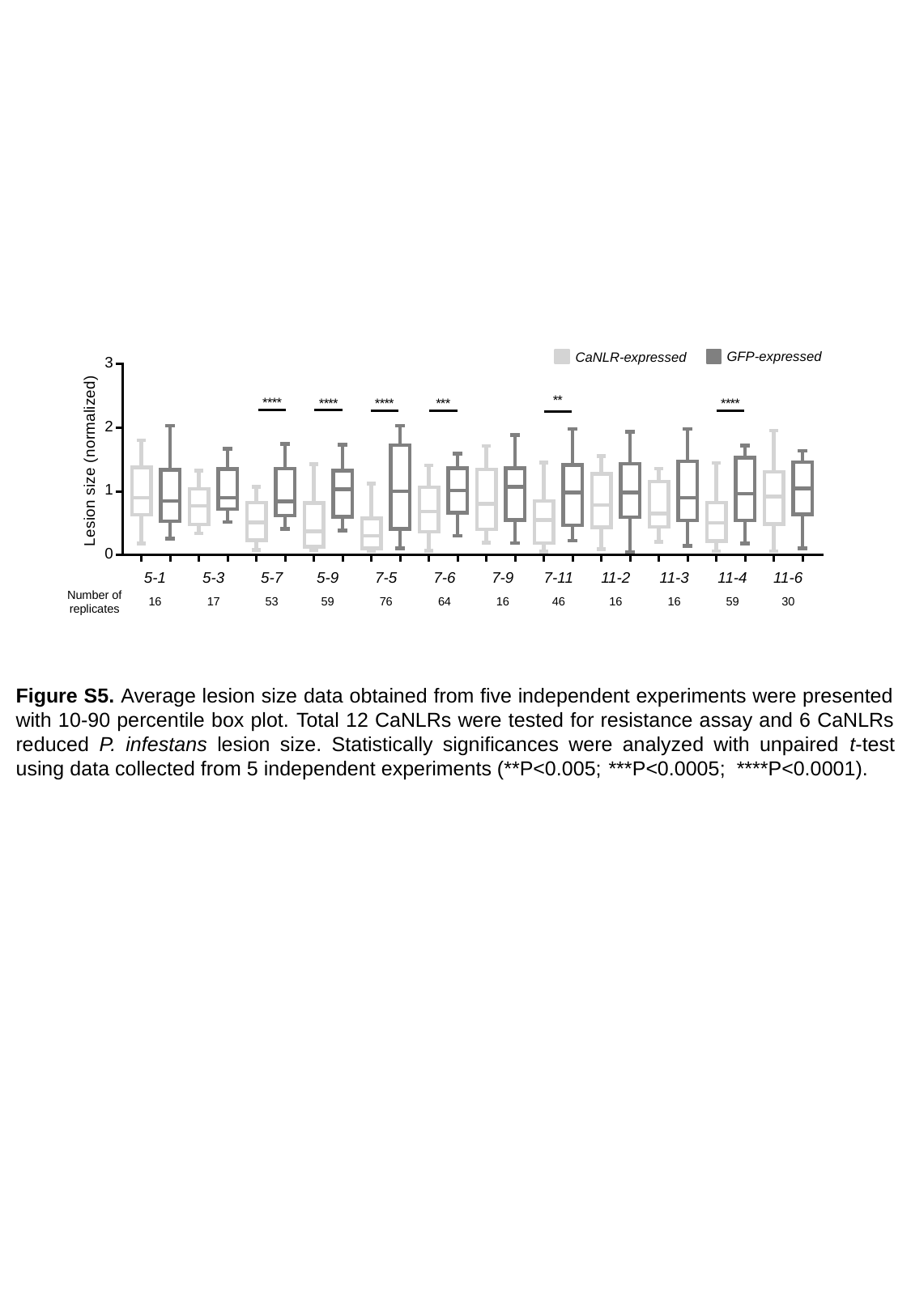

GFP-expressed
CaNLR-expressed
5-3
7-6
11-3
5-1
5-7
5-9
7-5
7-9
7-11
11-2
11-4
11-6
Number of
replicates
17
64
16
16
53
59
76
16
46
16
59
30
Figure S5. Average lesion size data obtained from five independent experiments were presented with 10-90 percentile box plot. Total 12 CaNLRs were tested for resistance assay and 6 CaNLRs reduced P. infestans lesion size. Statistically significances were analyzed with unpaired t-test using data collected from 5 independent experiments (**P<0.005; ***P<0.0005; ****P<0.0001).

## Slide 6
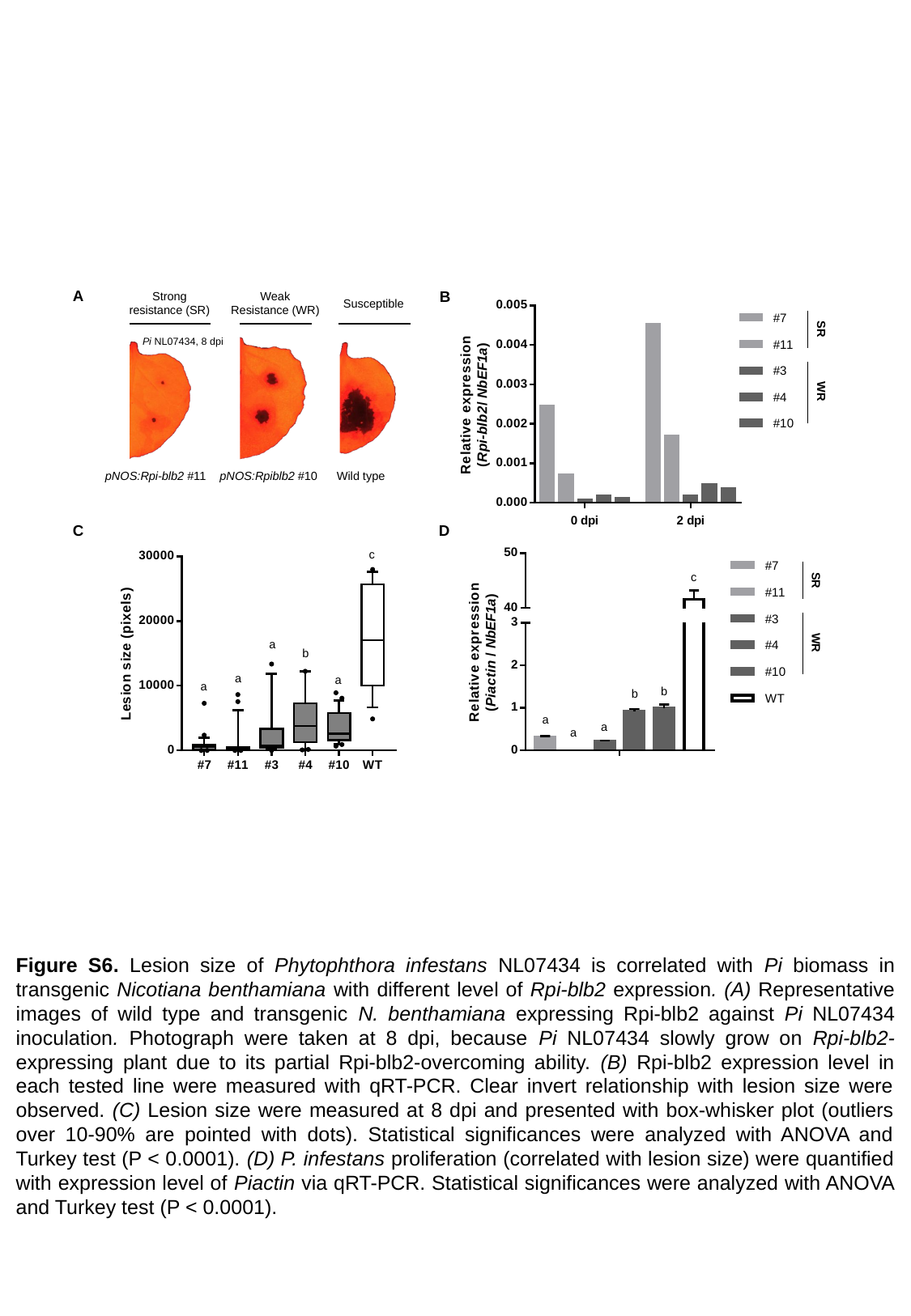

A
B
Strong
resistance (SR)
Weak
Resistance (WR)
Susceptible
SR
Pi NL07434, 8 dpi
WR
pNOS:Rpiblb2 #10
Wild type
pNOS:Rpi-blb2 #11
C
D
c
c
SR
WR
a
b
a
a
a
b
b
a
a
a
Figure S6. Lesion size of Phytophthora infestans NL07434 is correlated with Pi biomass in transgenic Nicotiana benthamiana with different level of Rpi-blb2 expression. (A) Representative images of wild type and transgenic N. benthamiana expressing Rpi-blb2 against Pi NL07434 inoculation. Photograph were taken at 8 dpi, because Pi NL07434 slowly grow on Rpi-blb2-expressing plant due to its partial Rpi-blb2-overcoming ability. (B) Rpi-blb2 expression level in each tested line were measured with qRT-PCR. Clear invert relationship with lesion size were observed. (C) Lesion size were measured at 8 dpi and presented with box-whisker plot (outliers over 10-90% are pointed with dots). Statistical significances were analyzed with ANOVA and Turkey test (P < 0.0001). (D) P. infestans proliferation (correlated with lesion size) were quantified with expression level of Piactin via qRT-PCR. Statistical significances were analyzed with ANOVA and Turkey test (P < 0.0001).

## Slide 7
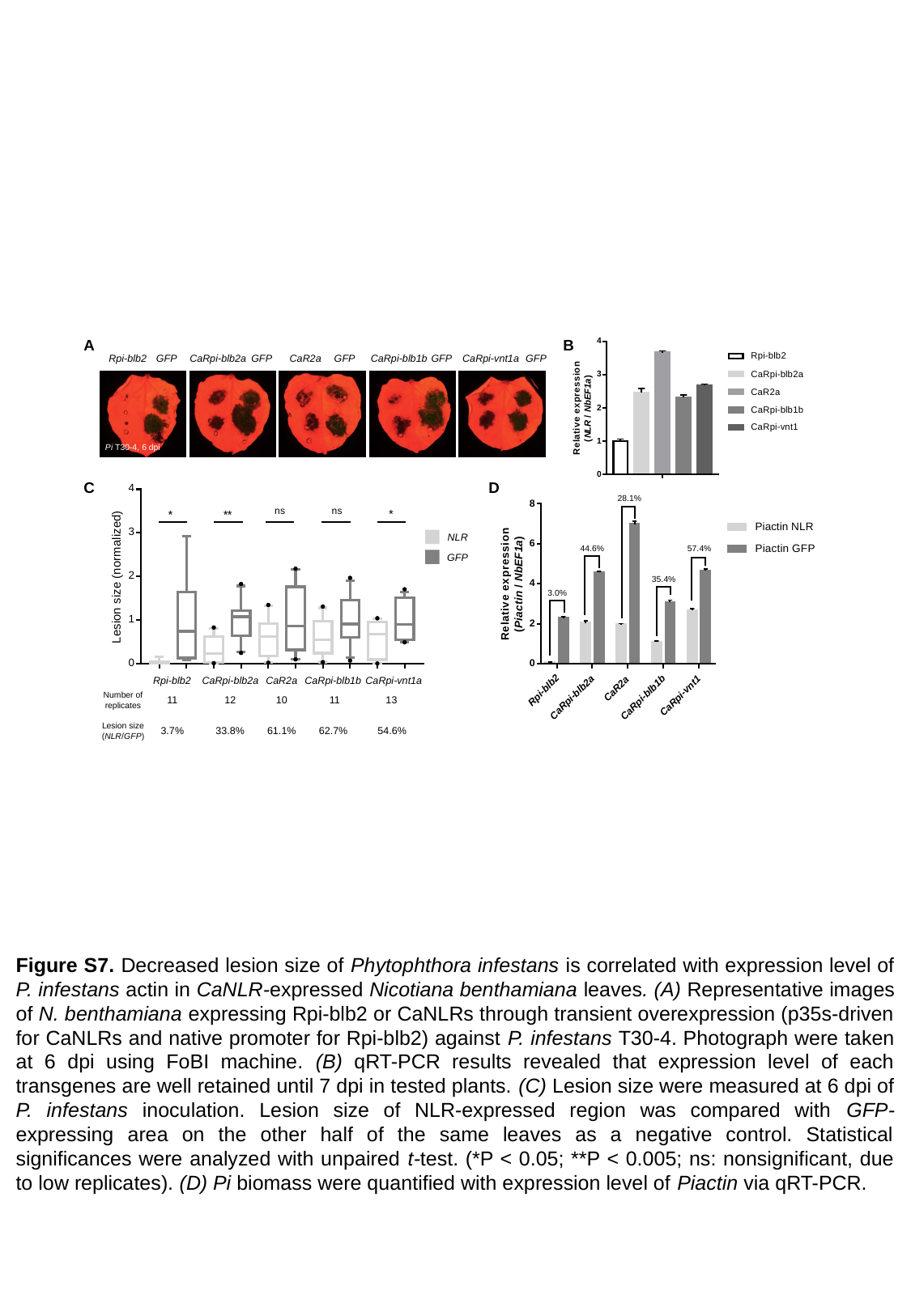

A
B
Rpi-blb2
GFP
CaRpi-blb2a
GFP
CaR2a
GFP
CaRpi-blb1b
GFP
CaRpi-vnt1a
GFP
Pi T30-4, 6 dpi
C
D
28.1%
NLR
57.4%
44.6%
GFP
35.4%
3.0%
Rpi-blb2
CaRpi-blb2a
CaR2a
CaRpi-blb1b
CaRpi-vnt1a
Number of
replicates
12
11
10
11
13
Lesion size
(NLR/GFP)
3.7%
33.8%
61.1%
62.7%
54.6%
Figure S7. Decreased lesion size of Phytophthora infestans is correlated with expression level of P. infestans actin in CaNLR-expressed Nicotiana benthamiana leaves. (A) Representative images of N. benthamiana expressing Rpi-blb2 or CaNLRs through transient overexpression (p35s-driven for CaNLRs and native promoter for Rpi-blb2) against P. infestans T30-4. Photograph were taken at 6 dpi using FoBI machine. (B) qRT-PCR results revealed that expression level of each transgenes are well retained until 7 dpi in tested plants. (C) Lesion size were measured at 6 dpi of P. infestans inoculation. Lesion size of NLR-expressed region was compared with GFP-expressing area on the other half of the same leaves as a negative control. Statistical significances were analyzed with unpaired t-test. (*P < 0.05; **P < 0.005; ns: nonsignificant, due to low replicates). (D) Pi biomass were quantified with expression level of Piactin via qRT-PCR.

## Slide 8
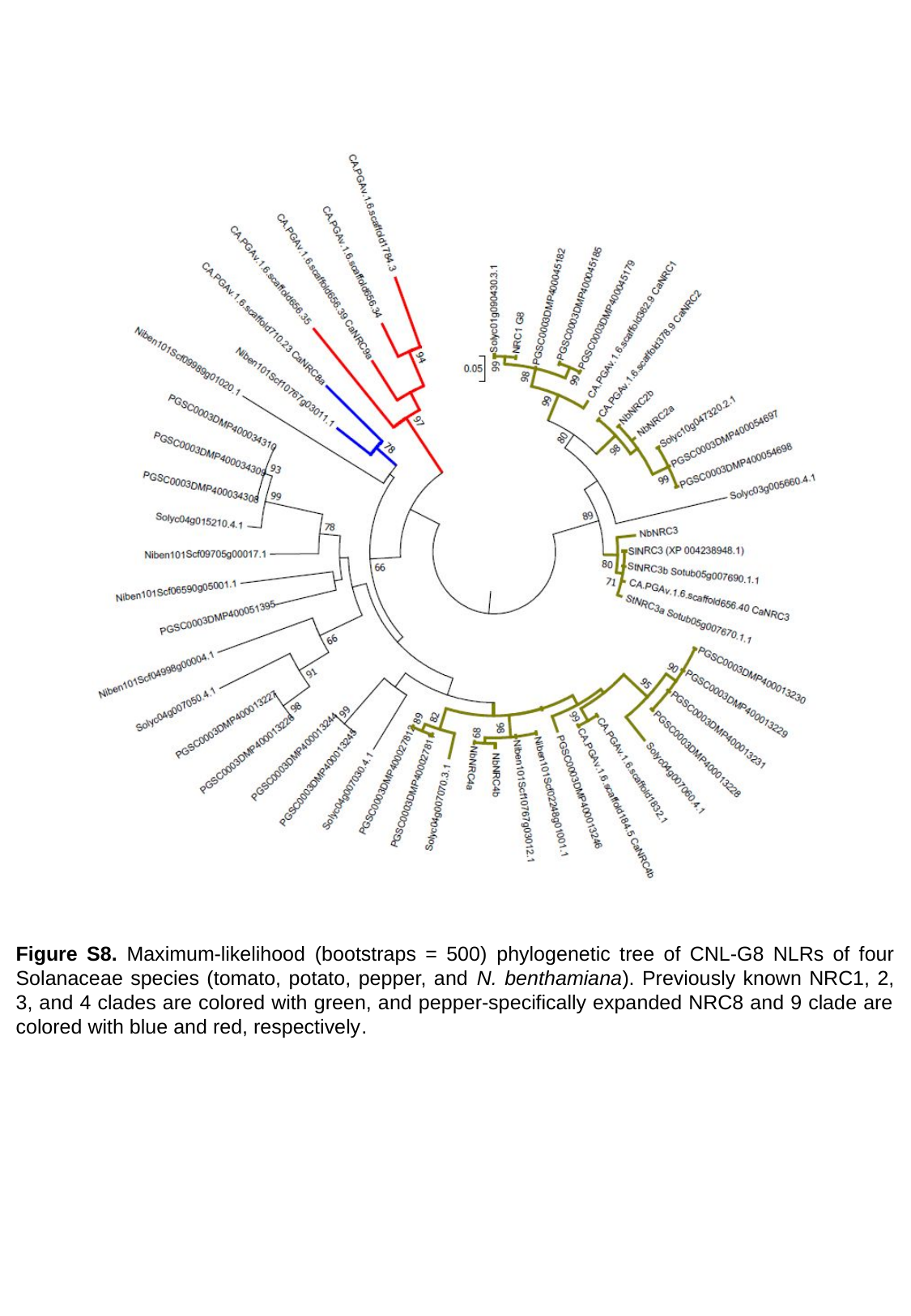

Figure S8. Maximum-likelihood (bootstraps = 500) phylogenetic tree of CNL-G8 NLRs of four Solanaceae species (tomato, potato, pepper, and N. benthamiana). Previously known NRC1, 2, 3, and 4 clades are colored with green, and pepper-specifically expanded NRC8 and 9 clade are colored with blue and red, respectively.

## Slide 9
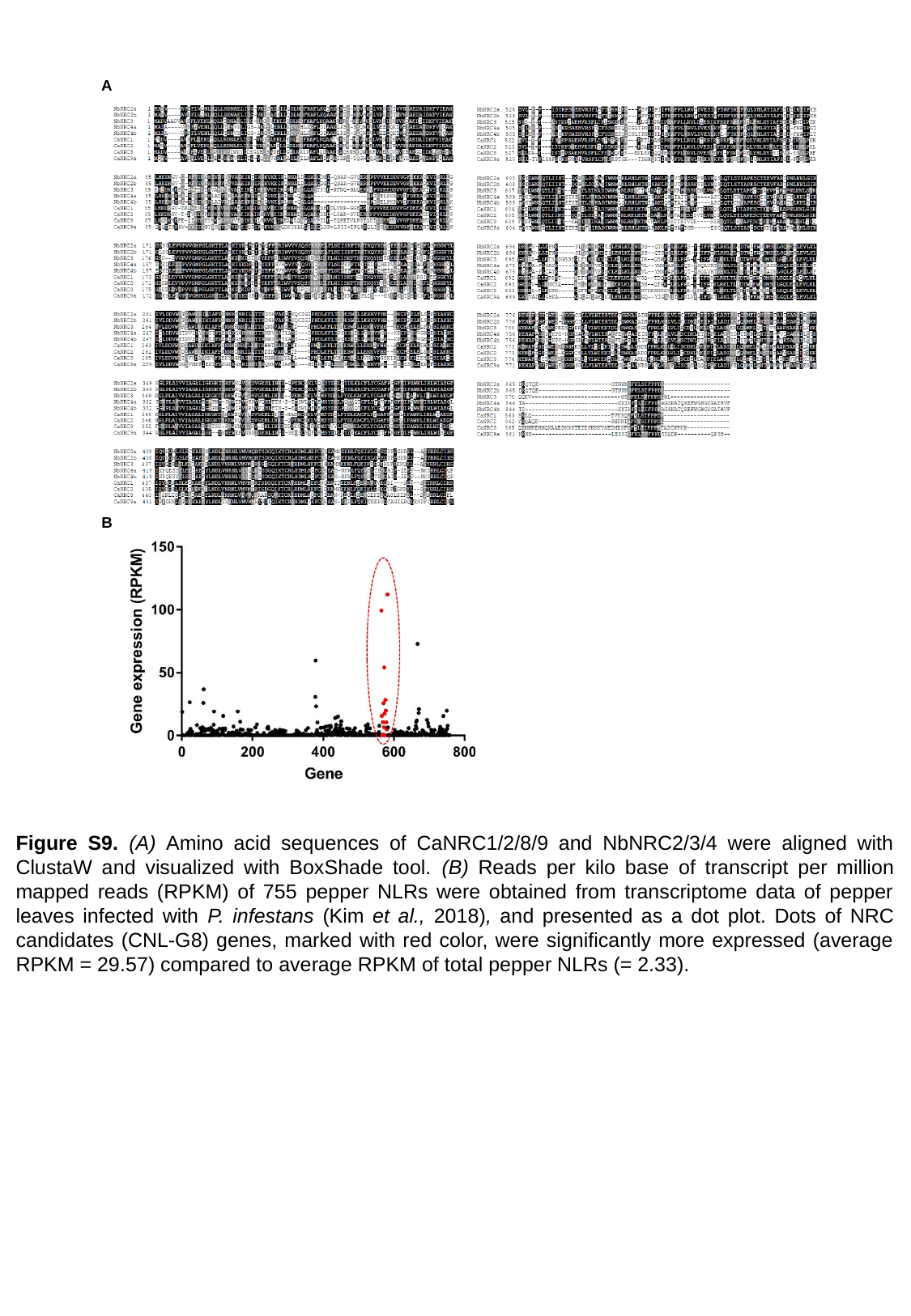

A
B
Figure S9. (A) Amino acid sequences of CaNRC1/2/8/9 and NbNRC2/3/4 were aligned with ClustaW and visualized with BoxShade tool. (B) Reads per kilo base of transcript per million mapped reads (RPKM) of 755 pepper NLRs were obtained from transcriptome data of pepper leaves infected with P. infestans (Kim et al., 2018), and presented as a dot plot. Dots of NRC candidates (CNL-G8) genes, marked with red color, were significantly more expressed (average RPKM = 29.57) compared to average RPKM of total pepper NLRs (= 2.33).

## Slide 10
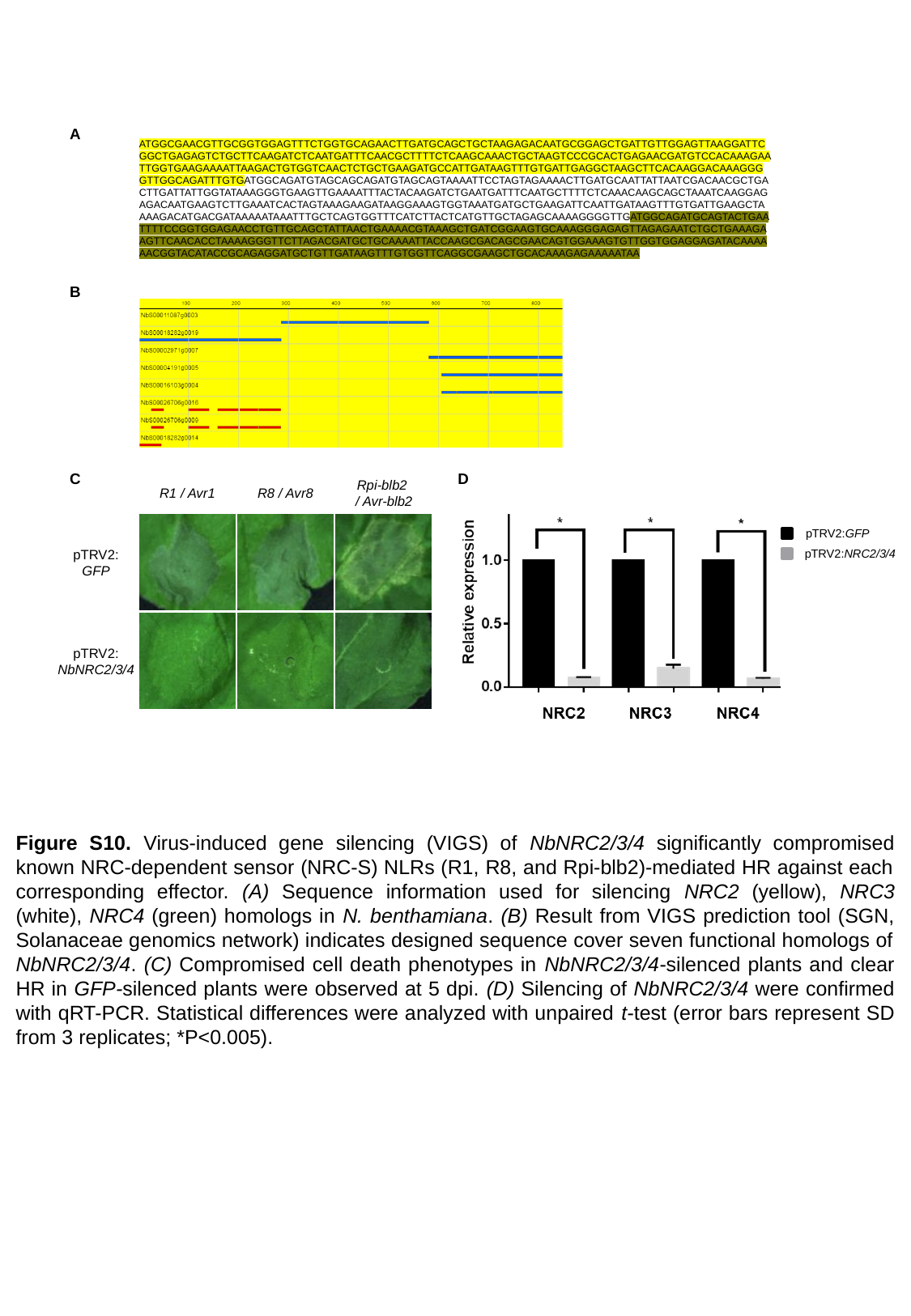

A
ATGGCGAACGTTGCGGTGGAGTTTCTGGTGCAGAACTTGATGCAGCTGCTAAGAGACAATGCGGAGCTGATTGTTGGAGTTAAGGATTCGGCTGAGAGTCTGCTTCAAGATCTCAATGATTTCAACGCTTTTCTCAAGCAAACTGCTAAGTCCCGCACTGAGAACGATGTCCACAAAGAATTGGTGAAGAAAATTAAGACTGTGGTCAACTCTGCTGAAGATGCCATTGATAAGTTTGTGATTGAGGCTAAGCTTCACAAGGACAAAGGGGTTGGCAGATTTGTGATGGCAGATGTAGCAGCAGATGTAGCAGTAAAATTCCTAGTAGAAAACTTGATGCAATTATTAATCGACAACGCTGACTTGATTATTGGTATAAAGGGTGAAGTTGAAAATTTACTACAAGATCTGAATGATTTCAATGCTTTTCTCAAACAAGCAGCTAAATCAAGGAGAGACAATGAAGTCTTGAAATCACTAGTAAAGAAGATAAGGAAAGTGGTAAATGATGCTGAAGATTCAATTGATAAGTTTGTGATTGAAGCTAAAAGACATGACGATAAAAATAAATTTGCTCAGTGGTTTCATCTTACTCATGTTGCTAGAGCAAAAGGGGTTGATGGCAGATGCAGTACTGAATTTTCCGGTGGAGAACCTGTTGCAGCTATTAACTGAAAACGTAAAGCTGATCGGAAGTGCAAAGGGAGAGTTAGAGAATCTGCTGAAAGAAGTTCAACACCTAAAAGGGTTCTTAGACGATGCTGCAAAATTACCAAGCGACAGCGAACAGTGGAAAGTGTTGGTGGAGGAGATACAAAAAACGGTACATACCGCAGAGGATGCTGTTGATAAGTTTGTGGTTCAGGCGAAGCTGCACAAAGAGAAAAATAA
B
C
D
Rpi-blb2
/ Avr-blb2
R8 / Avr8
R1 / Avr1
pTRV2:GFP
pTRV2:
GFP
pTRV2:NRC2/3/4
pTRV2:
NbNRC2/3/4
Figure S10. Virus-induced gene silencing (VIGS) of NbNRC2/3/4 significantly compromised known NRC-dependent sensor (NRC-S) NLRs (R1, R8, and Rpi-blb2)-mediated HR against each corresponding effector. (A) Sequence information used for silencing NRC2 (yellow), NRC3 (white), NRC4 (green) homologs in N. benthamiana. (B) Result from VIGS prediction tool (SGN, Solanaceae genomics network) indicates designed sequence cover seven functional homologs of NbNRC2/3/4. (C) Compromised cell death phenotypes in NbNRC2/3/4-silenced plants and clear HR in GFP-silenced plants were observed at 5 dpi. (D) Silencing of NbNRC2/3/4 were confirmed with qRT-PCR. Statistical differences were analyzed with unpaired t-test (error bars represent SD from 3 replicates; *P<0.005).

## Slide 11
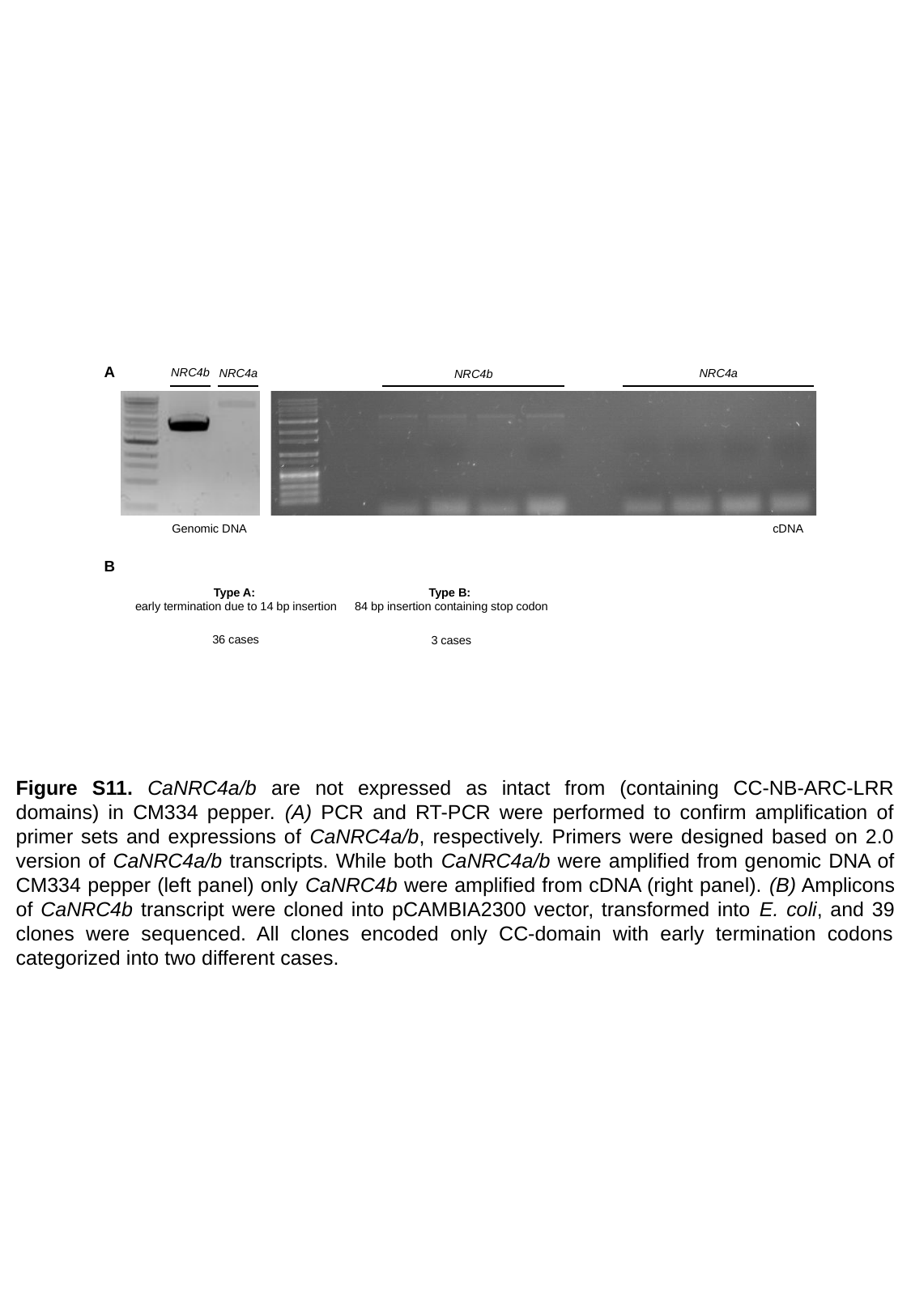

A
NRC4b
NRC4a
NRC4a
NRC4b
Genomic DNA
cDNA
B
Type A:
early termination due to 14 bp insertion
Type B:
84 bp insertion containing stop codon
36 cases
3 cases
Figure S11. CaNRC4a/b are not expressed as intact from (containing CC-NB-ARC-LRR domains) in CM334 pepper. (A) PCR and RT-PCR were performed to confirm amplification of primer sets and expressions of CaNRC4a/b, respectively. Primers were designed based on 2.0 version of CaNRC4a/b transcripts. While both CaNRC4a/b were amplified from genomic DNA of CM334 pepper (left panel) only CaNRC4b were amplified from cDNA (right panel). (B) Amplicons of CaNRC4b transcript were cloned into pCAMBIA2300 vector, transformed into E. coli, and 39 clones were sequenced. All clones encoded only CC-domain with early termination codons categorized into two different cases.

## Slide 12
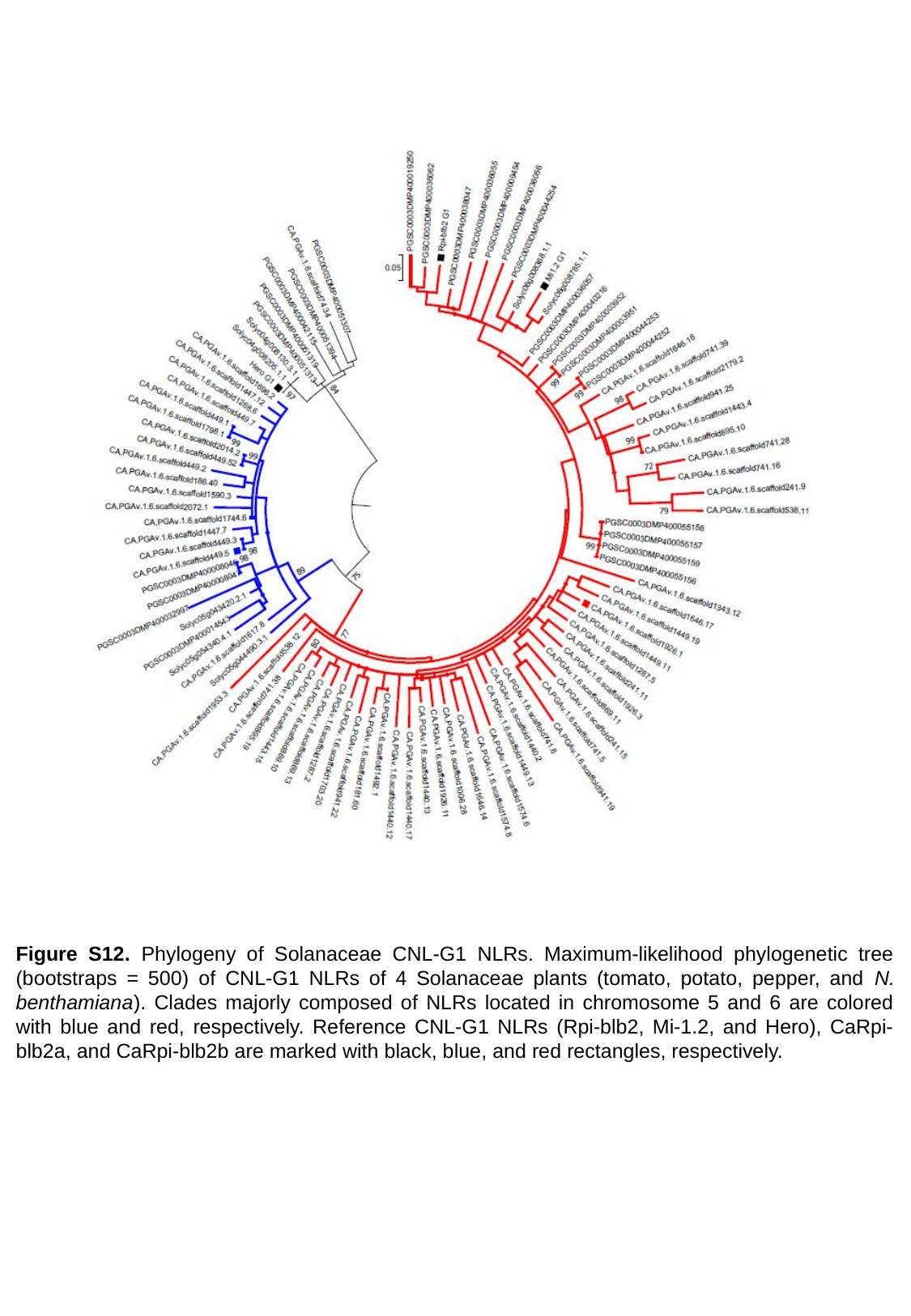

Figure S12. Phylogeny of Solanaceae CNL-G1 NLRs. Maximum-likelihood phylogenetic tree (bootstraps = 500) of CNL-G1 NLRs of 4 Solanaceae plants (tomato, potato, pepper, and N. benthamiana). Clades majorly composed of NLRs located in chromosome 5 and 6 are colored with blue and red, respectively. Reference CNL-G1 NLRs (Rpi-blb2, Mi-1.2, and Hero), CaRpi-blb2a, and CaRpi-blb2b are marked with black, blue, and red rectangles, respectively.

## Slide 13
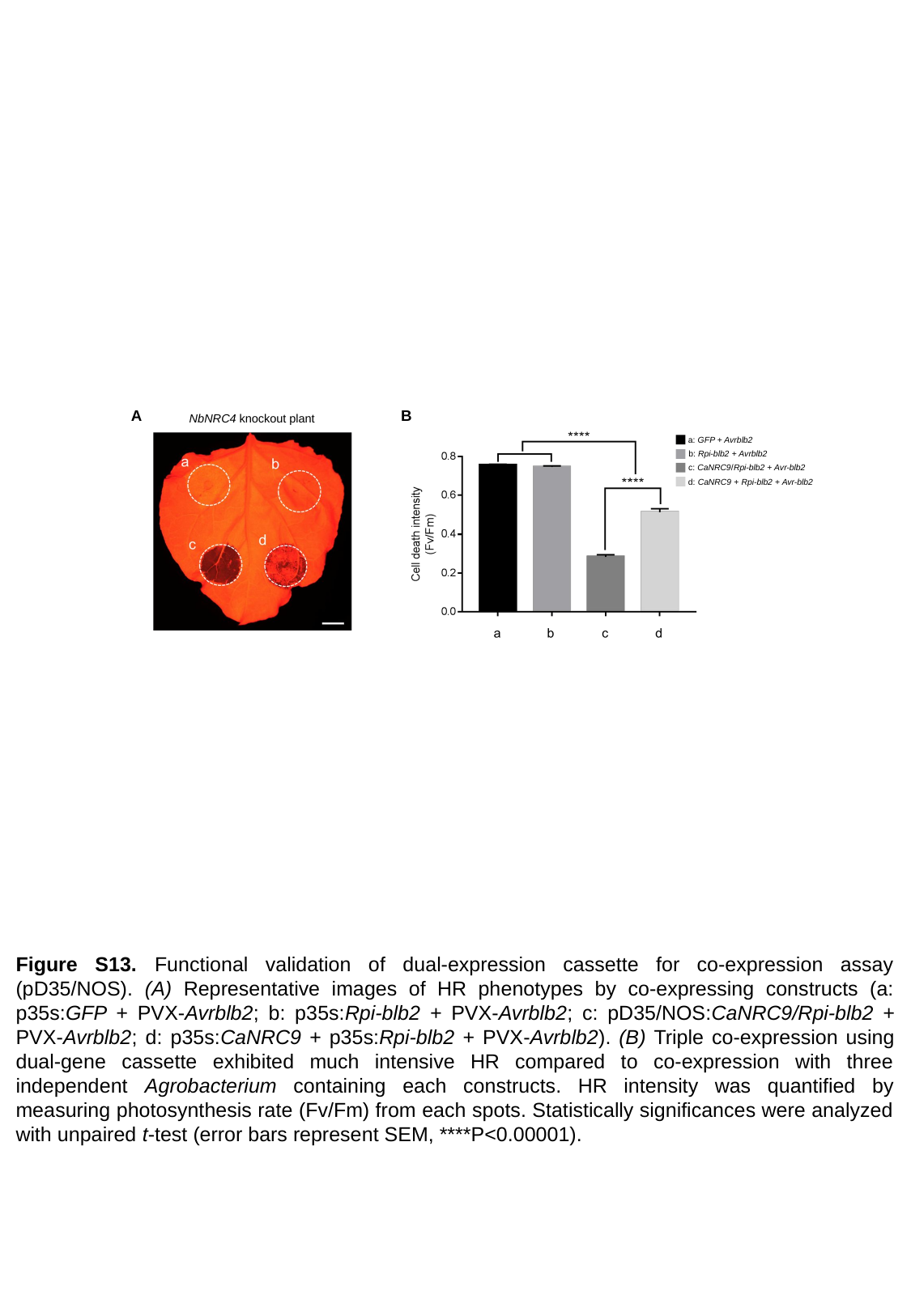

B
A
NbNRC4 knockout plant
a: GFP + Avrblb2
b: Rpi-blb2 + Avrblb2
c: CaNRC9/Rpi-blb2 + Avr-blb2
d: CaNRC9 + Rpi-blb2 + Avr-blb2
Figure S13. Functional validation of dual-expression cassette for co-expression assay (pD35/NOS). (A) Representative images of HR phenotypes by co-expressing constructs (a: p35s:GFP + PVX-Avrblb2; b: p35s:Rpi-blb2 + PVX-Avrblb2; c: pD35/NOS:CaNRC9/Rpi-blb2 + PVX-Avrblb2; d: p35s:CaNRC9 + p35s:Rpi-blb2 + PVX-Avrblb2). (B) Triple co-expression using dual-gene cassette exhibited much intensive HR compared to co-expression with three independent Agrobacterium containing each constructs. HR intensity was quantified by measuring photosynthesis rate (Fv/Fm) from each spots. Statistically significances were analyzed with unpaired t-test (error bars represent SEM, ****P<0.00001).

## Slide 14
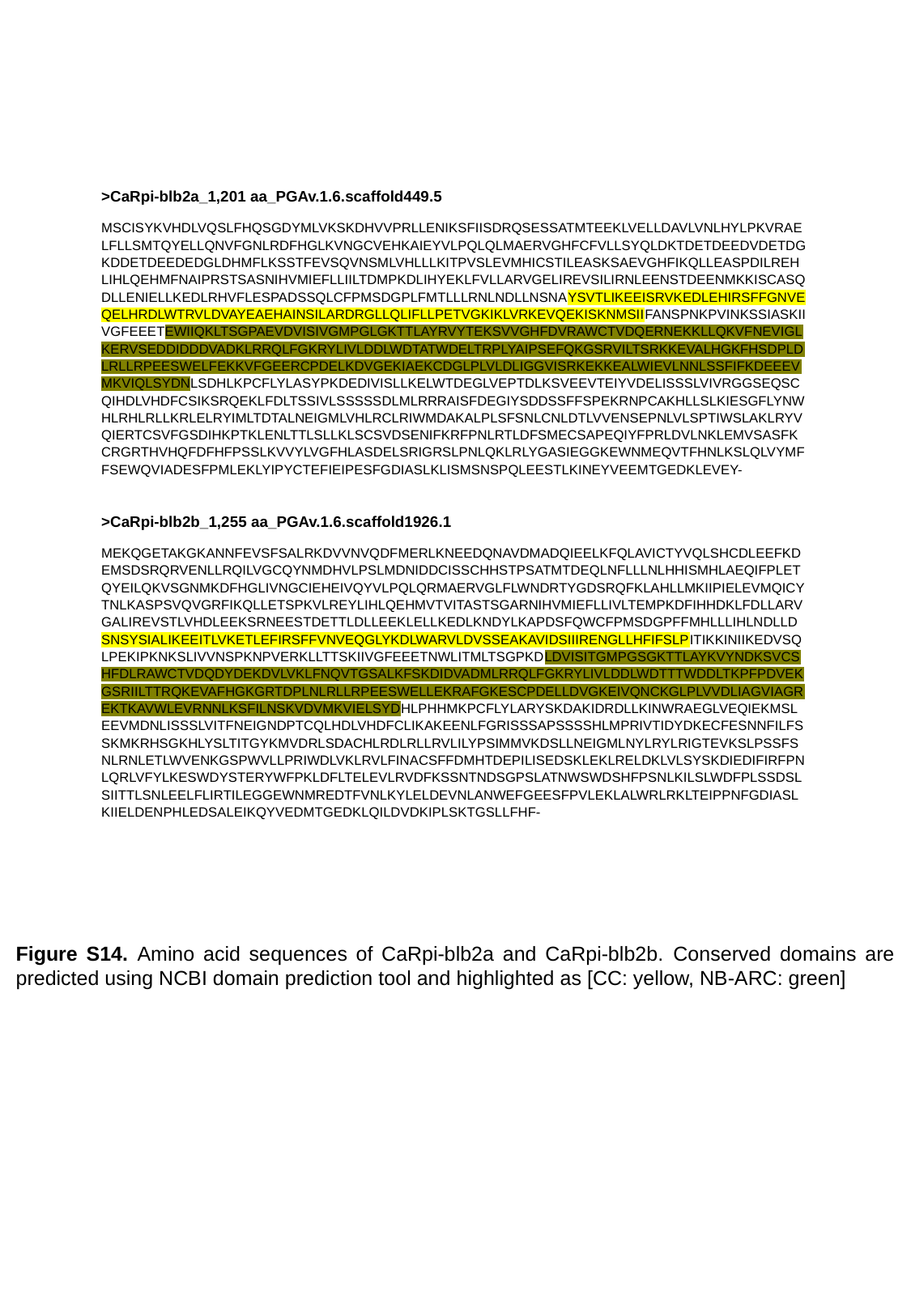

>CaRpi-blb2a_1,201 aa_PGAv.1.6.scaffold449.5
MSCISYKVHDLVQSLFHQSGDYMLVKSKDHVVPRLLENIKSFIISDRQSESSATMTEEKLVELLDAVLVNLHYLPKVRAELFLLSMTQYELLQNVFGNLRDFHGLKVNGCVEHKAIEYVLPQLQLMAERVGHFCFVLLSYQLDKTDETDEEDVDETDGKDDETDEEDEDGLDHMFLKSSTFEVSQVNSMLVHLLLKITPVSLEVMHICSTILEASKSAEVGHFIKQLLEASPDILREHLIHLQEHMFNAIPRSTSASNIHVMIEFLLIILTDMPKDLIHYEKLFVLLARVGELIREVSILIRNLEENSTDEENMKKISCASQDLLENIELLKEDLRHVFLESPADSSQLCFPMSDGPLFMTLLLRNLNDLLNSNAYSVTLIKEEISRVKEDLEHIRSFFGNVEQELHRDLWTRVLDVAYEAEHAINSILARDRGLLQLIFLLPETVGKIKLVRKEVQEKISKNMSIIFANSPNKPVINKSSIASKIIVGFEEETEWIIQKLTSGPAEVDVISIVGMPGLGKTTLAYRVYTEKSVVGHFDVRAWCTVDQERNEKKLLQKVFNEVIGLKERVSEDDIDDDVADKLRRQLFGKRYLIVLDDLWDTATWDELTRPLYAIPSEFQKGSRVILTSRKKEVALHGKFHSDPLDLRLLRPEESWELFEKKVFGEERCPDELKDVGEKIAEKCDGLPLVLDLIGGVISRKEKKEALWIEVLNNLSSFIFKDEEEVMKVIQLSYDNLSDHLKPCFLYLASYPKDEDIVISLLKELWTDEGLVEPTDLKSVEEVTEIYVDELISSSLVIVRGGSEQSCQIHDLVHDFCSIKSRQEKLFDLTSSIVLSSSSSDLMLRRRAISFDEGIYSDDSSFFSPEKRNPCAKHLLSLKIESGFLYNWHLRHLRLLKRLELRYIMLTDTALNEIGMLVHLRCLRIWMDAKALPLSFSNLCNLDTLVVENSEPNLVLSPTIWSLAKLRYVQIERTCSVFGSDIHKPTKLENLTTLSLLKLSCSVDSENIFKRFPNLRTLDFSMECSAPEQIYFPRLDVLNKLEMVSASFKCRGRTHVHQFDFHFPSSLKVVYLVGFHLASDELSRIGRSLPNLQKLRLYGASIEGGKEWNMEQVTFHNLKSLQLVYMFFSEWQVIADESFPMLEKLYIPYCTEFIEIPESFGDIASLKLISMSNSPQLEESTLKINEYVEEMTGEDKLEVEY-
>CaRpi-blb2b_1,255 aa_PGAv.1.6.scaffold1926.1
MEKQGETAKGKANNFEVSFSALRKDVVNVQDFMERLKNEEDQNAVDMADQIEELKFQLAVICTYVQLSHCDLEEFKDEMSDSRQRVENLLRQILVGCQYNMDHVLPSLMDNIDDCISSCHHSTPSATMTDEQLNFLLLNLHHISMHLAEQIFPLETQYEILQKVSGNMKDFHGLIVNGCIEHEIVQYVLPQLQRMAERVGLFLWNDRTYGDSRQFKLAHLLMKIIPIELEVMQICYTNLKASPSVQVGRFIKQLLETSPKVLREYLIHLQEHMVTVITASTSGARNIHVMIEFLLIVLTEMPKDFIHHDKLFDLLARVGALIREVSTLVHDLEEKSRNEESTDETTLDLLEEKLELLKEDLKNDYLKAPDSFQWCFPMSDGPFFMHLLLIHLNDLLDSNSYSIALIKEEITLVKETLEFIRSFFVNVEQGLYKDLWARVLDVSSEAKAVIDSIIIRENGLLHFIFSLPITIKKINIIKEDVSQLPEKIPKNKSLIVVNSPKNPVERKLLTTSKIIVGFEEETNWLITMLTSGPKDLDVISITGMPGSGKTTLAYKVYNDKSVCSHFDLRAWCTVDQDYDEKDVLVKLFNQVTGSALKFSKDIDVADMLRRQLFGKRYLIVLDDLWDTTTWDDLTKPFPDVEKGSRIILTTRQKEVAFHGKGRTDPLNLRLLRPEESWELLEKRAFGKESCPDELLDVGKEIVQNCKGLPLVVDLIAGVIAGREKTKAVWLEVRNNLKSFILNSKVDVMKVIELSYDHLPHHMKPCFLYLARYSKDAKIDRDLLKINWRAEGLVEQIEKMSLEEVMDNLISSSLVITFNEIGNDPTCQLHDLVHDFCLIKAKEENLFGRISSSAPSSSSHLMPRIVTIDYDKECFESNNFILFSSKMKRHSGKHLYSLTITGYKMVDRLSDACHLRDLRLLRVLILYPSIMMVKDSLLNEIGMLNYLRYLRIGTEVKSLPSSFSNLRNLETLWVENKGSPWVLLPRIWDLVKLRVLFINACSFFDMHTDEPILISEDSKLEKLRELDKLVLSYSKDIEDIFIRFPNLQRLVFYLKESWDYSTERYWFPKLDFLTELEVLRVDFKSSNTNDSGPSLATNWSWDSHFPSNLKILSLWDFPLSSDSLSIITTLSNLEELFLIRTILEGGEWNMREDTFVNLKYLELDEVNLANWEFGEESFPVLEKLALWRLRKLTEIPPNFGDIASLKIIELDENPHLEDSALEIKQYVEDMTGEDKLQILDVDKIPLSKTGSLLFHF-
Figure S14. Amino acid sequences of CaRpi-blb2a and CaRpi-blb2b. Conserved domains are predicted using NCBI domain prediction tool and highlighted as [CC: yellow, NB-ARC: green]

## Slide 15
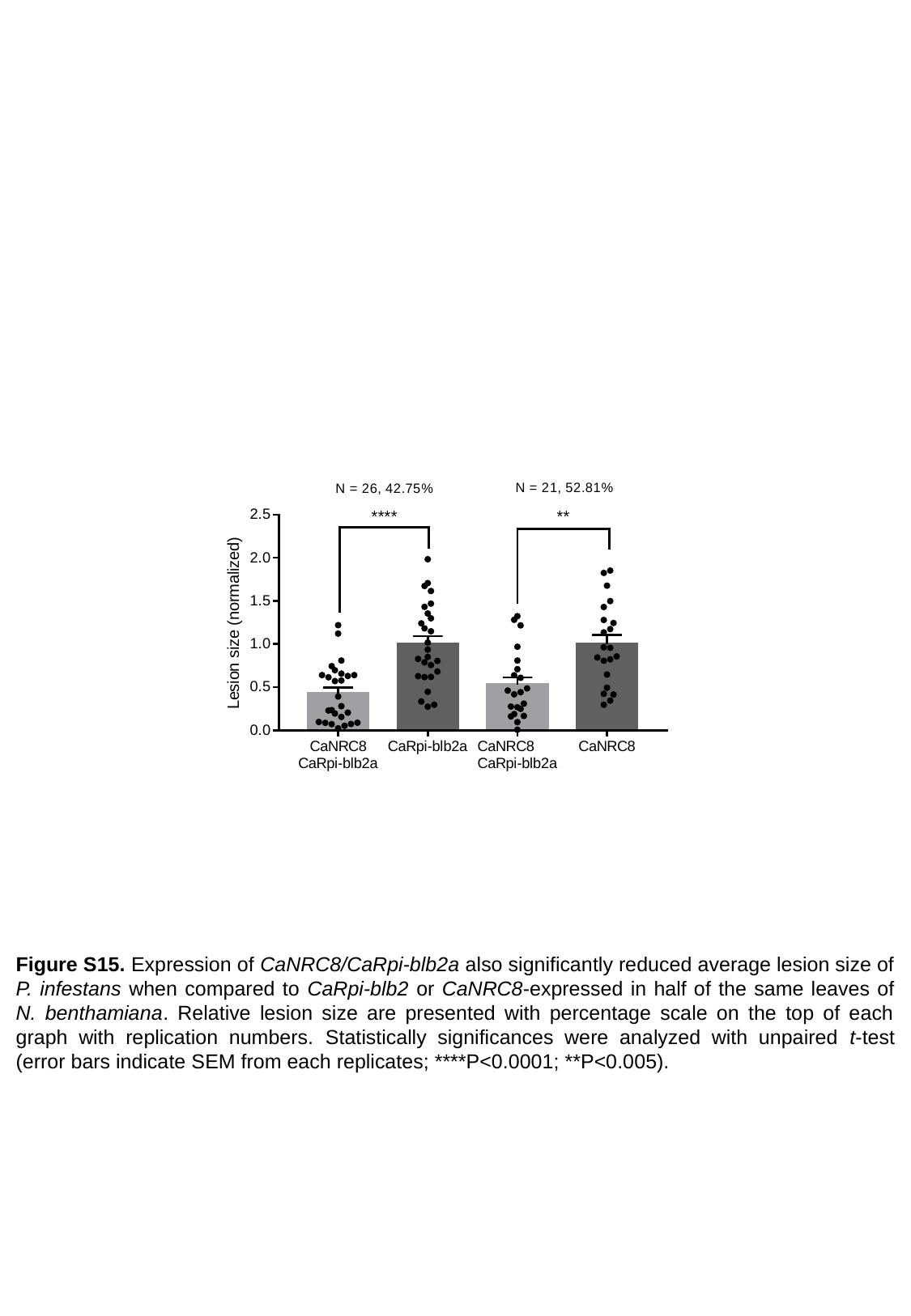

Figure S15. Expression of CaNRC8/CaRpi-blb2a also significantly reduced average lesion size of P. infestans when compared to CaRpi-blb2 or CaNRC8-expressed in half of the same leaves of N. benthamiana. Relative lesion size are presented with percentage scale on the top of each graph with replication numbers. Statistically significances were analyzed with unpaired t-test (error bars indicate SEM from each replicates; ****P<0.0001; **P<0.005).

## Slide 16
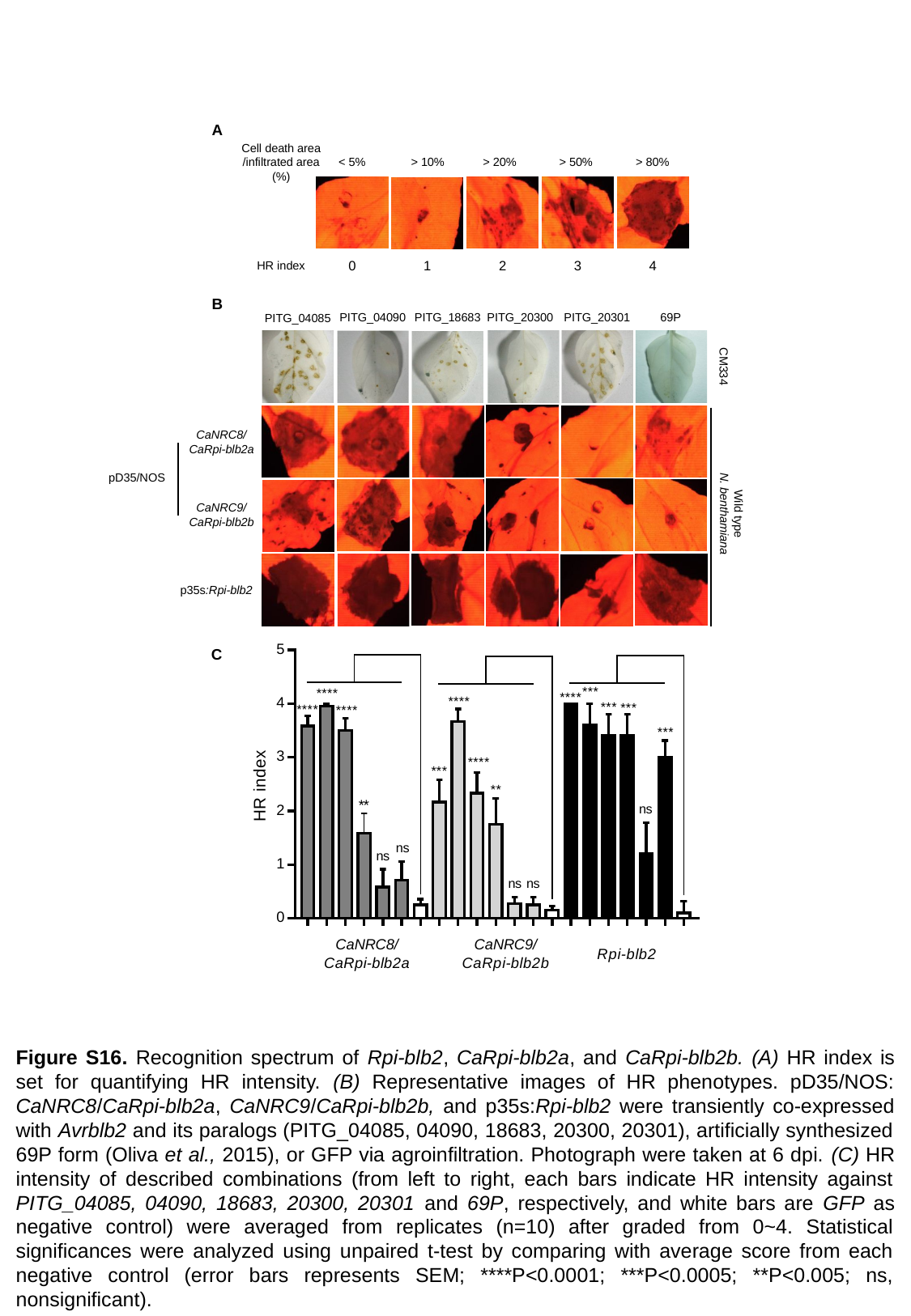

A
Cell death area
/infiltrated area
(%)
< 5%
> 10%
> 20%
> 50%
> 80%
0
1
2
3
4
HR index
B
PITG_18683
PITG_20301
69P
PITG_20300
PITG_04090
PITG_04085
CM334
CaNRC8/
CaRpi-blb2a
pD35/NOS
Wild type
N. benthamiana
CaNRC9/
CaRpi-blb2b
p35s:Rpi-blb2
C
Figure S16. Recognition spectrum of Rpi-blb2, CaRpi-blb2a, and CaRpi-blb2b. (A) HR index is set for quantifying HR intensity. (B) Representative images of HR phenotypes. pD35/NOS: CaNRC8/CaRpi-blb2a, CaNRC9/CaRpi-blb2b, and p35s:Rpi-blb2 were transiently co-expressed with Avrblb2 and its paralogs (PITG_04085, 04090, 18683, 20300, 20301), artificially synthesized 69P form (Oliva et al., 2015), or GFP via agroinfiltration. Photograph were taken at 6 dpi. (C) HR intensity of described combinations (from left to right, each bars indicate HR intensity against PITG_04085, 04090, 18683, 20300, 20301 and 69P, respectively, and white bars are GFP as negative control) were averaged from replicates (n=10) after graded from 0~4. Statistical significances were analyzed using unpaired t-test by comparing with average score from each negative control (error bars represents SEM; ****P<0.0001; ***P<0.0005; **P<0.005; ns, nonsignificant).

## Slide 17
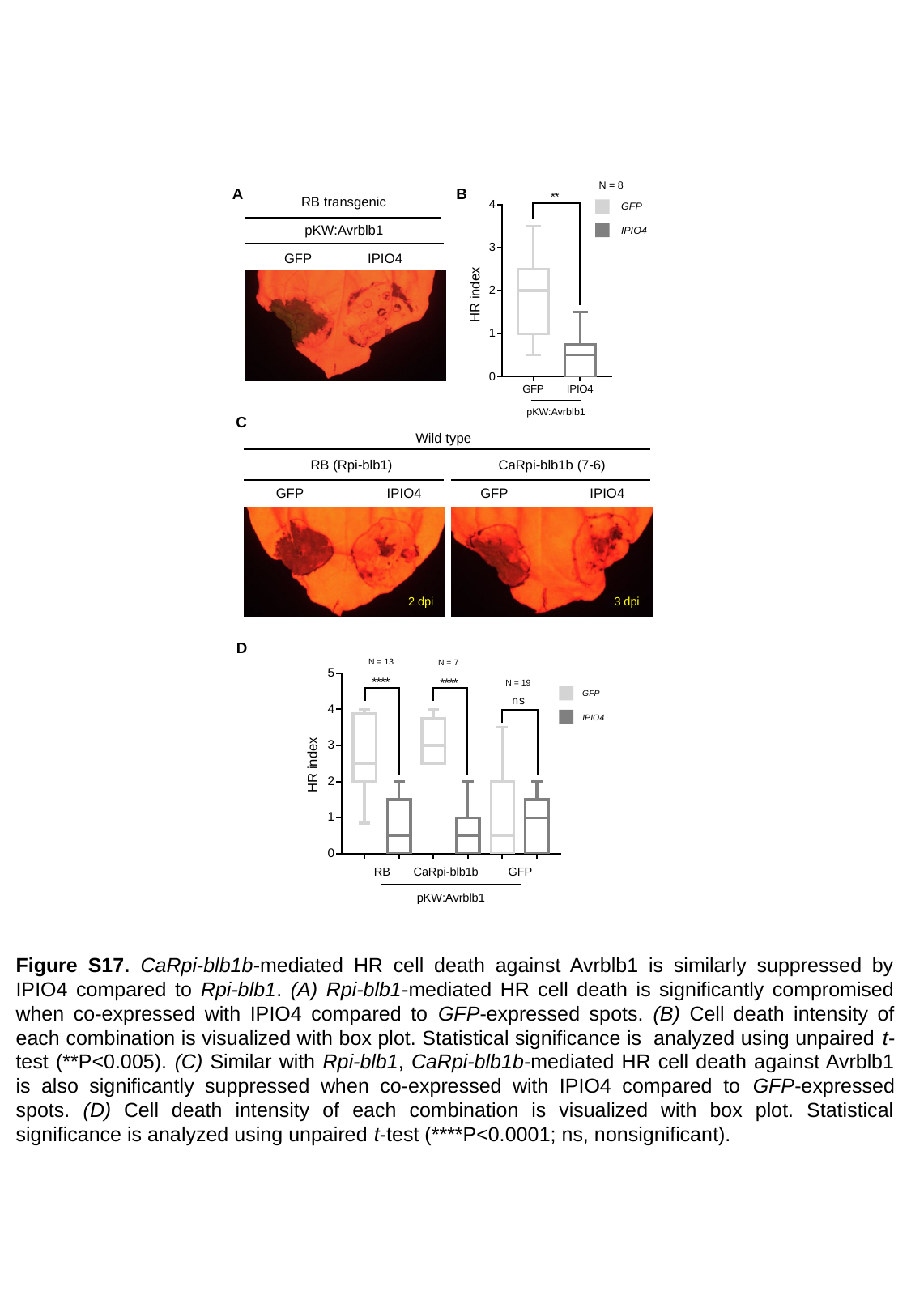

N = 8
A
B
RB transgenic
GFP
pKW:Avrblb1
IPIO4
GFP
IPIO4
HR index
pKW:Avrblb1
C
Wild type
RB (Rpi-blb1)
CaRpi-blb1b (7-6)
GFP
IPIO4
GFP
IPIO4
2 dpi
3 dpi
D
N = 13
N = 7
N = 19
GFP
IPIO4
HR index
CaRpi-blb1b
RB
GFP
pKW:Avrblb1
Figure S17. CaRpi-blb1b-mediated HR cell death against Avrblb1 is similarly suppressed by IPIO4 compared to Rpi-blb1. (A) Rpi-blb1-mediated HR cell death is significantly compromised when co-expressed with IPIO4 compared to GFP-expressed spots. (B) Cell death intensity of each combination is visualized with box plot. Statistical significance is analyzed using unpaired t-test (**P<0.005). (C) Similar with Rpi-blb1, CaRpi-blb1b-mediated HR cell death against Avrblb1 is also significantly suppressed when co-expressed with IPIO4 compared to GFP-expressed spots. (D) Cell death intensity of each combination is visualized with box plot. Statistical significance is analyzed using unpaired t-test (****P<0.0001; ns, nonsignificant).
