# Supplementary material for: Nucleotide‐binding leucine‐rich repeat network underlies nonhost resistance of pepper against the Irish potato famine pathogen Phytophthora infestans
Source: Plant Biotechnol J. 2023 Mar 13;21(7):1361–72. doi: 10.1111/pbi.14039 (PMC10281606; doi:10.1111/pbi.14039)
Supplement: Supplementary file 3 — Appendix S1 Materials and Methods. [file PBI-21-1361-s001.docx]

NLR network underlies nonhost resistance of pepper against the Irish potato famine pathogen *Phytophthora infestans*

Soohyun Oh^a,b^, Sejun Kim^a,b^, Hyo-Jeong Park^a,b^, Myung-Shin Kim^a^, Min-Ki Seo^a,b^, Chih-Hang Wu^c,f^, Hyun-Ah Lee^d^, Hyun-Soon Kim^e^, Sophien Kamoun^c^, and Doil Choi^a,b^

*Doil Choi

**Email:** [doil@snu.ac.kr](mailto:doil@snu.ac.kr)

**This PDF file includes:**

Supplementary text

SI References

Supplementary Information Text

SI Materials and Methods

***P. infestans* avirulence *e*ffector screening on pepper via agroinfiltration.** For additional identification of *Pi* effectors triggering cell death phenotypes on CM334 pepper, agrobacterium containing known *Pi* avirulence effectors (listed in Figure S2) were cultured for a day and re-suspended into infiltration buffer after spin down. OD_600_ were adjusted to 0.7 and infiltrated 3~4 weeks old CM334 pepper plants. Infiltrated plants were placed at growth chamber for 5~7 days. Cell death phenotypes were visualized under white / blue light conditions using Fluorescence *in vivo* Imaging system (FoBI, co, CELLGENTEK) machine. GFP was used as negative control. Cell death intensity were defined by measuring ratio of dead area / whole leaf area for each leaves using FoBI images.

**Co-expression assay of *Pi* avirulence effectors.** For identifying pepper NLRs recognizing *Avr1, Avr2, Avrblb1, Avrblb2,* or *Avrvnt1, agrobacterium* containing p35s:CaNLRs of each corresponding R genes (R1, R2, Rpi-blb1, Rpi-blb2, and Rpi-vnt1) for each avirulence effectors (listed in Table S2, S3, S4) were cultured for a day, and re-suspended into infiltration buffer after spin down. OD_600_ of NLRs and effectors were adjusted to 0.7 and 0.4, respectively and mixed into 1:1 ratio and infiltrated to 4-week old *N. benthamiana* leaves (pKW:GFP and p35s:GFP were used as negative control to test autoactive NLR-mediated cell death and effector-mediated weak cell death, respectively). Infiltrated plants were placed in growth chamber for 3~5 days (pepper NLRs co-expressed with Avr2, Avrblb1, and Avrvnt1 were scored at 5, 3, 4 dpi, respectively, because these three effectors exhibited cell death phenotypes expressed with GFP in *Nb* leaves after those timepoints (Table S1). Infiltrated leaves were detached and visualized under white / blue light condition using FoBI machine. We evaluated HR index of each cases and selected pepper NLRs exhibited significantly high scores when co-expressed with effectors compared to both controls.

**Resistance assay of CaNLRs recognizing *Pi* avirulence effectors.** Agrobacterium containing CaNLRs described in Figure S5 were cultured for a day and re-suspended into infiltration buffer after spin down. OD_600_ were adjusted to 0.3 and infiltrated half of 30-32 days old *N. benthamiana* leaves. GFP was expressed in on another half of the same leaves as a negative control. Infiltrated plants were placed in growth chamber for a day. Infiltrated leaves were detached and used for *Pi* T30-4 inoculation. Inoculated leaves were placed at at 21~23 ^o^C condition for 5~7 days. Lesion size were visualized under blue light using FoBI machine and measured with Image J program for statistical analyses.

**Quantifying *P. infestans* biomass and expression of NLRs in transient overexpression-based assay via qRT-PCR.** pNOS:*Rpi-blb2* transgenic lines and WT *N. benthamiana* plants were exploited for assay. Detached leaves of 4 weeks old Rpi-blb2 transgenic plants were inoculated by *Pi* NL07434 strains. Dead lesion size was measured at 8 dpi using imageJ program with FoBI image, and center of each inoculation site were sampled for qRT-PCR. RNA was isolated using TRIZOL (MRC®) reagent then used for cDNA synthesis with Suprescript III (Invitrogen®). qRT-PCR was performed using SYBRgreen supermix (BioRad®). p35s:CaNLRs and pNative:Rpi-blb2 were transiently overexpressed on 31 days old wild type *Nb* leaves via agroinfiltration. One day after agroinfiltration, *Pi* T30-4 strain was inoculated on detached leaves. Lesion size were measured at 6 dpi and center of each inoculated site were sampled for further procedure as describe above.

**Complementation assay of cell death mediated by *NRC*-dependent sensor NLRs.** *NbNRC2/3/4*-silenced *N. benthamiana* plants were exploited for cell death complementation assay. *Agrobacterium* suspensions containing previously reported *NRC*-dependent sensor (NRC-S) NLRs recognizing *P. infestans* RxLR effectors *R8* (6), and *Rpi-blb2* (7), were co-infiltrated with corresponding AVR effectors (*Avr8* (9), and *Avrblb2* (10), respectively) and NRC candidates of pepper (*CaNRC1, 2, 3, 4b, 8, 9a, 9b, 9c, 9d*) into *NbNRC2/3/4*-silenced (11) and *GFP*-silenced *N. benthamiana*. Each *Agrobacterium* suspensions containing *NRC-S*, *CaNRC*s, and effector constructs were mixed in 1:1:1 ratio after adjusted to OD_600_ of 0.6, 0.6, and 0.3, respectively. Cell death phenotypes were observed at 5 dpi under white light and blue light with yellow light filter condition using FoBI.

**Complementation assay of resistance against *P. infestans* mediated by *Rpi-blb2*.** *NbNRC2/3/4*-silenced pRpi-blb2:*Rpi-blb2*-transgenic *N. benthamiana* plants were exploited for resistance complementation assay. *Agrobacterium* suspensions containing *CaNRC8* or *CaNRC9a* were adjusted to OD_600_ of 0.3. Each half leaves of four-week-old *N. benthamiana* plants were infiltrated with one of described *Agrobacterium* suspensions and *Agrobacterium* containing p35s:*GFP* was infiltrated in another half as a negative control. Infiltrated leaves were detached at 1 dpi and inoculated with droplets of *Pi* T30-4 zoospore (10 μL, with 5.0 x 10^4^ spores/ml) on the abaxial side. Inoculated leaves were placed on wet tissue in SPL square plates and incubated in 21^o^C with 16 h light/8 h dark condition. Photographs were taken at 6 dpi under blue light with a yellow light filter using FoBI. Lesions sizes were measured using ImageJ program.

**Construction of dual gene expression cassette (pDual35/Nos).** Dual gene expression cassette (pD35/NOS) was constructed by modifying pCAMBIA2300-LIC vector as a backbone for efficient and consistent expression of multiple target genes. Pre-existed cloning site of p35s (cut ccdB selection marker with ApaI restriction enzyme; forward adapter: 5’-CGCCACAAGAGGCAGT-3’; reverse adapter: 5’-GCAAGACGAAGACAGT-3’) was modified, and additional LIC site with pNOS (cut mRFP selection marker with AatII restriction enzyme; forward adapter: 5’-GCGAGAGCAGGCGAGT-3’; reverse adapter: 5’-CGAGGACGACGCACGT-3’) site was inserted. Multiple cloning sites (MCS) containing variable restriction enzyme sites were also designed and inserted for the cases when target genes could be cleaved with ApaI or AatII.

**Triple co-expressions screening of pepper NLRs recognizing AVRblb2 (PITG_20300).** *Avrblb2* (PITG_20300) and *CaNRC8* or *CaNRC9a* were cloned into pD35/NOS vector (*CaNRC8*/*CaNRC9a* into p35s, and *Avrblb2* into pNOS site) for triple co-expression with 63 CNL-G1 sensor NLRs of pepper. *Agrobacterium* suspensions containing pD35/NOS:*CaNRC*/*Avrblb2* and each G1-NLRs of pepper were mixed in 1:1 ratio after adjusted to 0.5 of OD_600_. Four-week-old wild type and *NbNRC4*-knockout *N. benthamiana* (12) were infiltrated with *Agrobacterium* suspensions. Cell death images were taken at 7 dpi under white light and blue light with a yellow light filter using FoBI. The intensity of cell death phenotypes from 3 biological replicates was graded from 0 to 4 and averaged for quantification. Only *CaRpi-blb2a* (Ca05g17760) and *CaRpi-blb2b* (Ca00g87530) exhibited consistent and intensive (> 1 index) cell death phenotypes against *Avrblb2*. Thus, pD35/NOS:CaNRC8/CaRpi-blb2a and pD35NOS:CaNRC9/CaRpi-blb2b construct were additionally constructed for further examinations including cell death and resistance assays.

**Recognition spectrum analysis of *CaRpi-blb2a/b* against AVRblb2s and resistance to *P. infestans*.** *CaNRC8*/*CaRpi-blb2a* and *CaNRC9a*/*CaRpi-blb2b* pairs were cloned into pD35/NOS vector (*CaNRCs* into p35s, and NRC-S into pNOS site) for resistance test against *P. infestans* and triple co-expression with *Avrblb2* and its paralogs (PITG_20300, 04085, 04090, 18683, 20301), or artificially synthesized 69P (recognized by Rpi-blb2 but cannot trigger HR on pepper) form (10). i) For the resistance test, both NLR pairs were transiently expressed on a half leaf of 4-week-old NRC4-knockout *N. benthamiana* with *GFP* on another half as a negative control via agroinfiltration (OD_600_ of 0.3). Infiltrated leaves were detached at 1 dpi and inoculated with *Pi* T30-4 zoospore (10 μL, with 5.0 x 10^4^ spores/ml) on the abaxial side. Lesion sizes were measured using ImageJ program. Averaged lesion size from each NLR pair-expressed leaves was compared with averaged lesion size from the *GFP-*expressed half of the same leaves (after normalized to 1). Statistical significances were analyzed with unpaired t-test. ii) For recognition spectrum assay, *Agrobacterium* suspensions containing NLR pairs and Avrblb2 paralogs (including 69P form) were mixed in 1:1 ratio after adjusted to 0.7 for effectors and 0.4, respectively. Five-week-old *N. benthamiana* were infiltrated with mixed suspensions and placed at 22 ~ 25^o^C condition. Cell death images were taken at 6 dpi under white light and blue light with a yellow filter using FoBI. Cell death intensity was graded from 0 to 4 and averaged for quantification.

**Comparative suppression assay of Rpi-blb2, CaRpi-blb2a-mediated cell death against PITG_15278.** pD35/NOS:*CaNRC8/CaRpi-blb2a, CaNRC8/Rpi-blb2* were co-expressed with PVX-*Avrblb2* and p35s:*PITG_15278* or p35s:*GFP* in 3~4 week-old NRC4-knockout plants via agroinfiltration after adjusted to (OD_600_ of 0.5, 0.3, 0.4 respectively) and mixed in 1:1:1 ratio. Infiltrated plants were placed at 20 ~ 23^o^C condition. HR phenotypes of each triple co-expression with *PITG_15278* were averaged (graded from 0~4) and compared to co-expressed with *GFP* at 3~4 dpi*.* Statistical significances were analyzed with unpaired t-test.

**Comparative suppression assay of Rpi-blb2, CaRpi-blb2a-mediated resistance against *P. infestans* when co-expressed with PITG_15278.** pD35/NOS:*CaNRC8/CaRpi-blb2a, CaNRC8/Rpi-blb2* were co-expressed with pKW:*Avrblb2* and p35s:*PITG_15278* (13) or p35s:*GFP* in 3~4 week-old NRC4-knockout plants via agroinfiltration after adjusted to (OD_600_ of 0.3, 0.3 respectively) and mixed in 1:1 ratio. Infiltrated plants were placed at 22 ~ 24^o^C condition for a day. *Pi* T30-4 were inoculated on the abaxial side of each infiltrated leaves. Inoculated leaves were placed at 18 ^o^C chamber for a day and placed 21 ~ 22^o^C condition for 5~7 days. Lesion size were visualized with FoBI machine and measured with Image J program.

**Comparative suppression assay of Rpi-blb1, CaRpi-blb1a-mediated cell death against IPIO4.** p35s*:CaRpi-blb1a,* p35s:*Rpi-blb1* were co-expressed with p35s:*IPIO4* or p35s:*GFP* in 3~4 week-old NRC4-knockout plants via agroinfiltration after adjusted to (OD_600_ of 0.5, 0.4 respectively) and mixed in 1:1 ratio. Infiltrated plants were placed at 20 ~ 23^o^C condition for a day. pKW:*Avrblb1* containing agrobacterium were infiltrated on each previously infiltrated spots at 1 dpi. HR phenotypes of each triple co-expression spots were averaged (graded from 0~4) and compared to co-expressed with *GFP* at 3~4 dpi*.* Statistical significances were analyzed with unpaired t-test.

**SI References**

1. C. Koncz, J. Schell, The promoter of TL-DNA gene 5 controls the tissue-specific expression of chimaeric genes carried by a novel type of Agrobacterium binary vector. *Mol. Gen. Genet.* **204**, 383–396 (1986).

2. H. A. Lee, *et al.*, Multiple recognition of RXLR effectors is associated with nonhost resistance of pepper against *Phytophthora infestans*. *New Phytol.* **203**, 926–938 (2014).

3. E. Seo, S. Kim, S. Yeom, D. Choi, B. Petre, Genome-Wide comparative analyses reveal the dynamic evolution of repeat gene family among Solanaceae Plants. *Front. in Plant Sci.* **7**, 1–13 (2016).

4. H. Y. Lee, *et al.*, Genome-wide functional analysis of hot pepper immune receptors reveals an autonomous NLR clade in seed plants. *New Phytol.* **229**, 532–547 (2021).

5. A. Ballvora, *et al.*, The R1 gene for potato resistance to late blight (*Phytophthora infestans*) belongs to the leucine zipper/NBS/LRR class of plant resistance genes. *Plant J.* **30**, 361–371 (2002).

6. J. H. Vossen, *et al.*, The Solanum demissum R8 late blight resistance gene is an Sw-5 homologue that has been deployed worldwide in late blight resistant varieties. *Theor. Appl. Genet.* **129**, 1785–1796 (2016).

7. E. A. G. Van Der Vossen, *et al.*, The *Rpi-blb2* gene from Solanum bulbocastanum is an *Mi-1* gene homolog conferring broad-spectrum late blight resistance in potato. *Plant J.* **44**, 208–222 (2005).

8. Y. Du, M. H. Mpina, P. R. J. Birch, K. Bouwmeester, F. Govers, *Phytophthora infestans* RXLR effector AVR1 interacts with exocyst component Sec5 to manipulate plant immunity. *Plant Physiol.* **169**, 1975–1990 (2015).

9. K.-R. Jo, Unveiling and Deploying durability of late blight resistance in potato; From natural stacking to cisgenic stacking. *Ph. D. Thesis. Wageningen University, the Netherlands* (2013).

10. S. Oh, *et al.*, In Planta expression screens of *Phytophthora infestans* RXLR effectors reveal diverse phenotypes , including activation of the *Solanum bulbocastanum* disease resistance protein Rpi-blb2. *Plant Cell* **21**, 2928–2947 (2009).

11. C. H. Wu, *et al.*, NLR network mediates immunity to diverse plant pathogens. *Proc. Natl. Acad. Sci. U. S. A.* **114**, 8113–8118 (2017).

12. C. H. Wu, *et al.*, NRC4 gene cluster is not essential for bacterial flagellin-triggered immunity. *Plant Physiol.* **182**, 455–459 (2020).

13. L. Derevnina, *et al.*, Plant pathogens convergently evolved to counteract redundant nodes of an NLR immune receptor network. *PLoS Biol.* **19,** e3001136 (2021).

14. I. A. Shahmuradov, R. K. Umarov, V. V. Solovyev, TSSPlant: A new tool for prediction of plant Pol II promoters. *Nucleic Acids Res.* **45** (2017).
